# Silencing of STE20-type kinase TAOK1 confers protection against hepatocellular lipotoxicity through metabolic rewiring

**DOI:** 10.1097/HC9.0000000000000037

**Published:** 2023-03-17

**Authors:** Ying Xia, Emma Andersson, Sumit K. Anand, Emmelie Cansby, Mara Caputo, Sima Kumari, Rando Porosk, Kalle Kilk, Syam Nair, Hanns-Ulrich Marschall, Matthias Blüher, Margit Mahlapuu

**Affiliations:** 1 Department of Chemistry and Molecular Biology, Sahlgrenska University Hospital, University of Gothenburg, Gothenburg, Sweden; 2 Department of Biochemistry, Institute of Biomedicine and Translational Medicine, University of Tartu, Tartu, Estonia; 3 Institute of Neuroscience and Physiology, Institute of Clinical Sciences, Sahlgrenska Academy, University of Gothenburg, Gothenburg, Sweden; 4 Wallenberg Laboratory, Department of Molecular and Clinical Medicine, Institute of Medicine, Sahlgrenska University Hospital, University of Gothenburg, Gothenburg, Sweden; 5 Helmholtz Institute for Metabolic, Obesity, and Vascular Research (HI-MAG) of the Helmholtz Zentrum München, University Hospital Leipzig, University of Leipzig, Leipzig, Germany

## Abstract

NAFLD has become the leading cause of chronic liver disease worldwide afflicting about one quarter of the adult population. NASH is a severe subtype of NAFLD, which in addition to hepatic steatosis connotes liver inflammation and hepatocyte ballooning. In light of the exponentially increasing prevalence of NAFLD, it is imperative to gain a better understanding of its molecular pathogenesis. The aim of this study was to examine the potential role of STE20-type kinase TAOK1 —a hepatocellular lipid droplet-associated protein—in the regulation of liver lipotoxicity and NAFLD etiology.

**Methods:**

The correlation between *TAOK1* mRNA expression in liver biopsies and the severity of NAFLD was evaluated in a cohort of 62 participants. Immunofluorescence microscopy was applied to describe the subcellular localization of TAOK1 in human and mouse hepatocytes. Metabolic reprogramming and oxidative/endoplasmic reticulum stress were investigated in immortalized human hepatocytes, where TAOK1 was overexpressed or silenced by small interfering RNA, using functional assays, immunofluorescence microscopy, and colorimetric analysis. Migration, invasion, and epithelial-mesenchymal transition were examined in TAOK1-deficient human hepatoma-derived cells. Alterations in hepatocellular metabolic and pro-oncogenic signaling pathways were assessed by immunoblotting.

**Results:**

We observed a positive correlation between the TAOK1 mRNA abundance in human liver biopsies and key hallmarks of NAFLD (*i.e.*, hepatic steatosis, inflammation, and ballooning). Furthermore, we found that TAOK1 protein fully colocalized with intracellular lipid droplets in human and mouse hepatocytes. The silencing of TAOK1 alleviated lipotoxicity in cultured human hepatocytes by accelerating lipid catabolism (mitochondrial β-oxidation and triacylglycerol secretion), suppressing lipid anabolism (fatty acid influx and lipogenesis), and mitigating oxidative/endoplasmic reticulum stress, and the opposite changes were detected in TAOK1-overexpressing cells. We also found decreased proliferative, migratory, and invasive capacity, as well as lower epithelial-mesenchymal transition in TAOK1-deficient human hepatoma-derived cells. Mechanistic studies revealed that TAOK1 knockdown inhibited ERK and JNK activation and repressed acetyl-CoA carboxylase (ACC) protein abundance in human hepatocytes.

**Conclusions:**

Together, we provide the first experimental evidence supporting the role of hepatic lipid droplet-decorating kinase TAOK1 in NAFLD development through mediating fatty acid partitioning between anabolic and catabolic pathways, regulating oxidative/endoplasmic reticulum stress, and modulating metabolic and pro-oncogenic signaling.

## INTRODUCTION

NAFLD is emerging as the leading cause of chronic liver disease, afflicting ~25% of the global population.^1^,^2^ As a hepatic manifestation of metabolic syndrome, NAFLD is frequently associated with obesity, dyslipidemia, and type 2 diabetes.^1^ Excessive fat accumulation within hepatocytes is considered a key event in the initiation of NAFLD.^2^ As the disease advances, a subset of NAFLD patients progress to NASH, which in addition to hepatic steatosis is characterized by local inflammation and cell damage, carrying an increased risk of developing liver fibrosis, cirrhosis, and HCC.^3^ Thus, deciphering the molecular mechanisms underlying the initiation and aggravation of NAFLD is of high clinical importance to develop strategies for its prevention and management.

In NAFLD, hydrophobic neutral lipids [primarily triacylglycerols (TAGs) and cholesteryl esters] accumulate within intrahepatocellular lipid droplets (LDs), covered by a monolayer of phospholipids and associated proteins.^4^ Notably, the best-characterized genetic risk factors controlling the susceptibility of NAFLD—*PNPLA3* and *HSD17B13*—both encode proteins anchored to the hepatic LDs.^5^,^6^ Moreover, our recent studies have provided several lines of evidence that various STE20-type kinases—STK25, MST3, MST4, and TAOK3—bind to LDs and critically regulate the dynamic balance of lipid storage versus lipid utilization within the liver, contributing to the pathogenesis of NAFLD.^7^–^16^ Consequently, hepatic LD-associated proteins have emerged as potential targets for combating NAFLD and related metabolic disorders.

We recently identified thousand and one kinase 1 (TAOK1; also known as MAP3K16 or PSK2) as a hepatic LD-binding protein based on a global proteomic analysis of LD fraction isolated from livers of high-fat diet-fed mice.^10^,^15^ TAOK1 belongs to the STE20 kinase family and is implicated in a range of functions in different cell types. TAOK1 has been reported to regulate p38 mitogen-activated protein kinase (MAPK)-mediated DNA damage responses by interacting with MEK3 in human cervical carcinoma cell line,^17^,^18^ to induce apoptotic morphological alterations by stimulating the JUN N-terminal kinase (JNK) pathway in human non–small cell lung carcinoma cell line (H1299),^19^ and to restrict cell proliferation by phosphorylating Hippo core components in human embryonic kidney cell line (HEK293).^20^ In addition, oxidative stress was found to enhance TAOK1 protein levels in human hepatocytes, and microRNA miR-706 directly inhibiting TAOK1 expression was shown to alleviate liver fibrosis in mice.^21^ Overexpression of TAOK1 has also been detected in a wide range of cancers including breast, colorectal, and lung cancer, as well as HCC.^22^

On the basis of its subcellular localization around hepatic LDs, we here hypothesized that TAOK1 may contribute to the molecular pathogenesis of human NAFLD. By combining gene expression analysis in liver biopsies with *in vitro* investigations in cultured hepatocytes, we provide the first evidence suggesting a possible role of TAOK1 in the development and progression of NAFLD through mediating fatty acid channeling between anabolic and catabolic pathways, regulating oxidative/endoplasmic reticulum (ER) stress, and modulating metabolic and pro-oncogenic signaling pathways.

## MATERIALS AND METHODS

### Analysis of liver biopsies of human participants

The *TAOK1* mRNA expression was determined in liver biopsies from 62 White individuals (men, n=35; women, n=27) who were recruited from subjects undergoing laparoscopic abdominal surgery for Roux-en-Y bypass (n=12), sleeve gastrectomy (n=9), or elective cholecystectomy (n=41). Total body fat was analyzed by dual x-ray absorptiometry and liver fat content was assessed by single-proton magnetic resonance spectroscopy (1H-MRS), as previously described.^23^ After the overnight withdrawal of food, liver biopsies were collected during the surgery (between 08:00 and 10:00 am), immediately snap frozen in liquid nitrogen, and stored at −80°C for further preparation. In human liver biopsies, histological features were blindly evaluated by 2 specialized hepatopathologists in hematoxylin and eosin—and Oil Red O-stained sections using the well-validated NAFLD activity score (NAS), as recommended by the NASH Clinical Research Network classification system.^24^ Quantitative real-time PCR (qRT-PCR) analysis on liver biopsies was performed, as described below using the probes for *TAOK1* (Hs01020477_m1) and 18S rRNA (Hs99999901_s1; Thermo Fisher Scientific, Waltham, MA), which span exon-exon boundaries to improve the specificity. For participant characteristics and details on inclusion/exclusion criteria, see Cansby et al.^10^

All investigations were approved by the Ethics Committee of the University of Leipzig, Germany (approval numbers 363-10-13122010 and 159-12-21052012) and conducted in compliance with both the Declarations of Helsinki and Istanbul. All patients enrolled in this study voluntarily provided written consent to use their anonymized data.

### Cell culture and transient transfections

Immortalized human hepatocytes (IHHs; a kind gift from B. Staels, the Pasteur Institute of Lille, University of Lille Nord de France, Lille, France), HepG2-NTCP cells (human hepatoma-derived cells; a kind gift from S. Urban, Department of Infectious Diseases, University Hospital Heidelberg, Heidelberg, Germany), and LX-2 cells (human stellate cells; Millipore, Burlington, MA) were cultured and maintained, as previously described.^16^,^25^,^26^ THP-1 cells (human monocytic cells; American Type Culture Collection, Manassas, VA) were cultured and differentiated into macrophages induced by the treatment with 100 nmol/L phorbol 12-myristate 13-acetate (PMA; Sigma-Aldrich, St. Louis, MO) for 48 hours.

For RNA interference, cultured human hepatocytes and liver nonparenchymal cells were transfected with human *TAOK1* small interfering (si)RNA (M-004846-03; Dharmacon, Lafayette, CO) or scrambled siRNA (D-001206-13; Dharmacon) using Lipofectamine RNAiMax (Thermo Fisher Scientific). For overexpression, IHHs were transfected with human *MYC*-tagged *TAOK1* expression plasmid (EX-T7024-M43; GeneCopoeia, Nivelles, Belgium) or an empty control plasmid (EX-NEG-M43; GeneCopoeia) using Lipofectamine 2000 (Thermo Fisher Scientific). Twenty-four hours after transfections, the culture medium was replaced by fresh medium, with or without supplementation of 100 µmol/L oleic acid (Sigma-Aldrich), for subsequent 48-hour incubation (Supplemental Figure S1, http://links.lww.com/HC9/A120).

### Assessment of lipid metabolism and oxidative/ER stress

To quantify neutral lipids, mitochondrial activity/content, and superoxide radicals, cells were stained with Bodipy 493/503 (Invitrogen, Carlsbad, CA), MitoTracker Red or Green (Thermo Fisher Scientific), or dihydroethidium (DHE; Life Technologies, Grand Island, NY), respectively. In parallel, cells were processed for immunofluorescence with anti-TAOK1, anti-adipose differentiation-related protein (ADRP), anti-LC3, anti-8-oxoguanine (8-oxoG), anti-4-hydroxynonenal (4-HNE), anti-E06, anti-KDEL, anti-C/EBP-homologous protein (CHOP), anti-peroxisomal biogenesis factor 5 (PEX5), or anti-peroxisomal membrane protein 70 kDa (PMP70) antibodies (Supplemental Table S1, http://links.lww.com/HC9/A121 for antibody information). Immunofluorescence images were acquired using a Zeiss Axio Observer microscope with the ZEN Blue software (Zeiss, Oberkochen, Germany). The labeled area was quantified in 6 randomly selected microscopic fields (×20) per well of the cell culture chamber using the ImageJ software (1.47v; National Institutes of Health, Bethesda, MD). Intracellular hydrogen peroxide (H_2_O_2_) and oxidative damage to proteins were detected using the dichlorodihydrofluorescein diacetate (DCFDA)/H2DCFDA-Cellular ROS Assay Kit (Abcam, Cambridge, UK) and the Protein Carbonyl Content Assay Kit (Sigma-Aldrich), respectively, according to the manufacturer’s instructions.

Liver sections from mice fed a high-fat diet (45 kcal% fat; D12451; Research Diets, New Brunswick, NJ) were processed for immunofluorescence with anti-TAOK1, anti-ADRP, anti-glial fibrillary acidic protein (GFAP), or anti-F4/80 antibodies (Supplemental Table S1, http://links.lww.com/HC9/A121, for antibody information).

β-oxidation, TAG secretion, incorporation of media-derived [^3^H]glucose and [^3^H]oleic acid into TAGs, and TAG hydrolase activity were measured in human hepatocytes, as described in the Supporting Materials http://links.lww.com/HC9/A206. The formation of both autophagosome and autolysosome was detected using the Premo Autophagy Tandem Sensor RFP-GFP-LC3B Kit (Thermo Fischer Scientific), according to the manufacturer’s instructions.

### Measurement of glucose metabolism

Glycogen levels, glucose uptake, the rate of glycogenolysis, gluconeogenesis, basal glycolysis, and compensatory glycolysis were assessed in IHHs, as described in the Supporting Materials http://links.lww.com/HC9/A206.

### Evaluation of proliferation, apoptosis, migration, invasion, and epithelial-mesenchymal transition

The proliferation of HepG2-NTCP cells was analyzed using the Click-iT EdU Proliferation Assay for Microplates Kit (Thermo Fisher Scientific), according to the manufacturer’s instructions. The Apoptosis/Necrosis Detection Kit (Abcam) was applied to monitor the initial/intermediate stages of apoptosis by staining with Apopxin Green for phosphatidylserine. The activation of caspase 3 and caspase 7 was determined using the Caspase-Glo 3/7 Assay Kit (Promega, Stockholm, Sweden) following the manufacturer’s protocol. The capacity of migration and invasion and epithelial-mesenchymal transition were also assessed in HepG2-NTCP cells as described in the Supporting Materials, http://links.lww.com/HC9/A206.

### qRT-PCR, coimmunoprecipitation, and western blot

RNA was isolated from tissue samples and cultured human hepatocytes and the following cDNA synthesis was performed, as described in the Supporting Materials, http://links.lww.com/HC9/A206. Coimmunoprecipitation was carried out using anti-MYC (Anti-c-MYC Magnetic Beads; Thermo Fisher Scientific) or anti-FLAG (Anti-FLAG M2 Magnetic Beads; Sigma-Aldrich) antibodies, according to the manufacturer’s instructions. Western blot analysis was performed as described previously^27^ (Supplemental Table S1, http://links.lww.com/HC9/A121, for antibody information).

### Statistical analysis

Statistical significance between the groups was evaluated using the unpaired 2-tailed Student *t* test with a value of *p*<0.05 considered statistically significant. Correlation between *TAOK1* expression in human liver biopsies and hepatic lipid content, as well as NAS was examined by Spearman rank correlation analysis after the Kolmogorov-Smirnov test assessing normality of data. All statistical analyses were performed using SPSS statistics (v27; IBM Corporation, Armonk, NY).

## RESULTS

### Association between hepatic TAOK1 expression and the severity of NAFLD in humans

**Figure 1:**
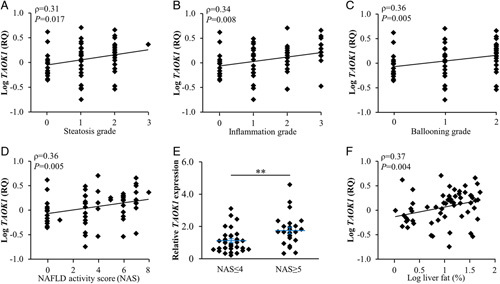
Hepatic *TAOK1* expression is significantly and positively correlated with the severity of NAFLD. (A-D) Correlation between *TAOK1* mRNA abundance determined in human liver biopsies by qRT-PCR and the severity of the individual components of NAS (liver steatosis, inflammation, hepatocellular ballooning; A-C) as well as composite NAS (D). (E) Hepatic *TAOK1* transcript levels in subjects with low versus high NAS (NAS≤4 versus NAS≥5, respectively). (F) Correlation between hepatic *TAOK1* mRNA expression and liver fat content measured by magnetic resonance spectroscopy (^1^H-MRS). **p<0.01. Abbreviations: NAS, NAFLD activity score; RQ, relative quantification; TAOK1, thousand and one kinase 1.

The pathological grade of NAFLD is clinically determined in liver biopsies using the NAS, which is composed of the histological scores of the severity of liver steatosis, lobular inflammation, and hepatocyte ballooning.^24^ Thus, we first analyzed the hepatic *TAOK1* mRNA expression in relation to the NAS in a cohort of 62 participants with a wide range in BMI (22.7–45.6 kg/m^2^), body fat (19.5%–57.9%), and liver fat (1.1%–50.0%). We found a positive correlation between *TAOK1* levels and all 3 features of the NAS, as well as the composite NAS (Figure 1A–D). In addition, we observed that the patient subset presenting NAS ≥5 (indicates definitive NASH; n=24) had a slight but significant increase in *TAOK1* abundance compared with the patient subset presenting NAS ≤4 (indicates simple steatosis or borderline NASH; n=35) (Figure 1E). Consistent with the analysis of histological steatosis score, we detected a positive correlation between the hepatic *TAOK1* transcript and liver fat content measured by magnetic resonance spectroscopy (Figure 1F). We found no association between *TAOK1* expression and sex, waist-to-hip ratio, BMI, or whole blood HbA1c values of the participants; however, hepatic *TAOK1* levels correlated positively with body fat (Supplemental Figure S2, http://links.lww.com/HC9/A120).

### TAOK1 coats intrahepatocellular LDs and is abundant in liver nonparenchymal cells

**Figure 2:**
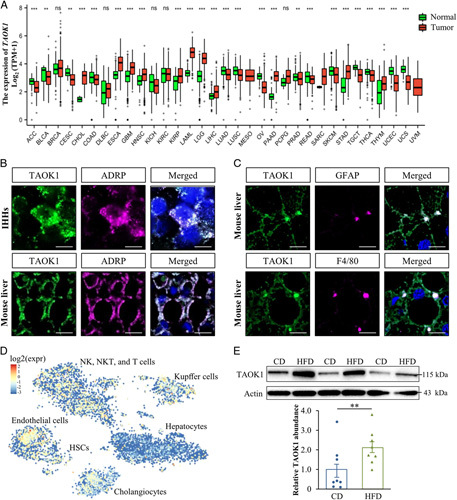
TAOK1 coats intracellular LDs in hepatocytes and is abundant in liver nonparenchymal cells. (A) Gene expression of TAOK1 in 30 normal and 33 carcinoma tissue types from the Genotype-Tissue Expression Portal and the Cancer Genome Atlas. Cancer tissues are shown in red and the corresponding normal tissues are shown in green. The box plots show the median (line in a box), first-to-third quartiles (boxes), 1.5× the interquartile range (whiskers), and outer (dots). (B) Representative images of oleate-treated IHHs and liver sections from high-fat diet-fed mice, double-stained with antibodies for TAOK1 (green) and ADRP (violet); merged image shows colocalization in white; nuclei stained with DAPI (blue). The scale bars at the top and bottom represent 7.5 and 15 µm, respectively. (C) Representative images of liver sections from high-fat diet-fed mice double-stained with anti-TAOK1 (green) and anti-GFAP or anti-F4/80 (violet) antibodies; merged image shows colocalization in white; nuclei stained with DAPI (blue). The scale bars represent 15 µm. (D) Distribution of *TAOK1* expression in human liver determined by single-cell sequencing data from the Human Liver Cell Atlas. (E) Liver lysates from mice fed with a high-fat diet for 20 weeks, and age-matched chow-fed controls, were assessed by western blot. Protein levels were analyzed by densitometry; representative western blots are shown with actin used as a loading control. Data are mean±SEM from 8 mice per group. **p<0.01, ***p<0.001. Abbreviations: ADRP, adipose differentiation-related protein; CD, chow diet; expr, expression; GFAP, glial fibrillary acidic protein; HFD, high-fat diet; HSCs, hepatic stellate cells; IHHs, immortalized human hepatocytes; NK, natural killer; ns, not significant; NTC, nontargeting control; TAOK1, thousand and one kinase 1.

Earlier studies by Northern blot have suggested a ubiquitous expression of human *TAOK1*.^28^ Consistently, the analysis of data available in the Genotype-Tissue Expression Portal and the Cancer Genome Atlas demonstrated the presence of *TAOK1* in a broad range of human normal tissues and carcinoma types including the liver and HCC samples (Figure 2A; HCC designated as liver HCC, LIHC). Our previous studies using global proteomics by liquid chromatography–mass spectrometry technique detected TAOK1 in the LD fraction isolated from livers of obese mice.^10^,^15^ However, the differences between *bona-fide* LD proteins and contaminating proteins are difficult to determine by proteomic studies, since LDs are closely associated with a wide range of membrane-bound cellular organelles.^4^ To this end, we here further investigated the subcellular localization of endogenous TAOK1 in cultured human hepatocytes and in liver sections from high-fat diet-fed mice using immunofluorescence microscopy. We confirmed that TAOK1 protein is concentrated on the surface of intrahepatocellular LDs, visualized by ADRP staining (Figure 2B). Importantly, ADRP is the main LD-coating protein, which enables highly specific labeling of LD as it is rapidly degraded in the absence of LD binding.^29^ We also found that TAOK1 is abundant in liver nonparenchymal cells identified by immunostaining for GFAP (marker of hepatic stellate cells) or F4/80 (macrophage marker) (Figure 2C). Similarly, single-cell sequencing data from the Human Liver Cell Atlas showed that within the liver, *TAOK1* is expressed in Kupffer cells and HSCs but even in endothelial cells, cholangiocytes, natural killer NK cells, NKT cells, and T cells, in addition to hepatocytes (Figure 2D). Interestingly, both western blot and immunofluorescence microscopy analysis demonstrated that the TAOK1 protein level was notably increased in the livers from mice fed a high-fat diet compared with age-matched chow-fed controls (Figure 2E; Supplemental Figure S3, http://links.lww.com/HC9/A120).

### TAOK1 controls hepatocellular lipid partitioning

**Figure 3:**
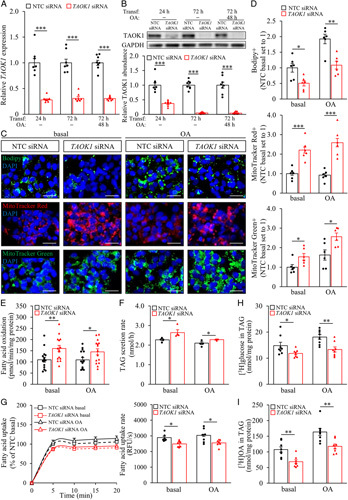
The silencing of TAOK1 stimulates lipid catabolism and suppresses lipid anabolism in human hepatocytes. IHHs were transfected with *TAOK1* siRNA or NTC siRNA and cultured with or without oleate supplementation as indicated. TAOK1 mRNA (A) and protein (B) abundance were assessed by qRT-PCR and western blot, respectively. In (B), protein levels were analyzed by densitometry; representative western blots are shown with glyceraldehyde-3-phosphate dehydrogenase (GAPDH) used as a loading control. Representative images of cells stained with Bodipy (green), MitoTracker Red (red), or MitoTracker Green (green); nuclei stained with DAPI (blue) (C). The scale bars represent 20 µm. (D) Quantification of the staining. (E) Oxidation of radiolabeled palmitate. (F) Secretion of [^3^H]TAG into the media. (G) Fatty acid uptake rate. TAG synthesis from [^3^H]-labeled glucose (H) and [^3^H]-labeled oleic acid (I). Data are mean±SEM from 4–8 (A–D and F–I) or 15 (E) wells per group. *p<0.05, **p<0.01, ***p<0.001. Abbreviations: GAPDH, glyceraldehyde-3-phosphate dehydrogenase; NTC, nontargeting control; OA, oleic acid; TAG, triacylglycerol; TAOK1, thousand and one kinase 1; Transf, transfection.

Hepatocellular lipotoxicity is widely recognized as an initiating pathology in NAFLD/NASH.^4^ Thus, we analyzed the effect of modifying the abundance of TAOK1 on intracellular lipid accumulation in cultured human hepatocytes. For TAOK1 knockdown, IHHs were transfected with *TAOK1*-specific siRNA or with a nontargeting control (NTC) siRNA; in parallel to the experiments carried out under basal culture conditions, cells were also treated with oleic acid to replicate the environment of high-risk individuals. As expected, TAOK1 mRNA and protein expression was significantly diminished in IHHs transfected with *TAOK1* siRNA (Figure 3A, B). Immunostainings with anti-TAOK1 antibody also demonstrated a high transfection efficacy in hepatocytes (Supplemental Figure S4, http://links.lww.com/HC9/A120).

First, we stained the transfected cells with the lipophilic dye Bodipy 493/503 to measure the amount of neutral lipids. Quantification of Bodipy-positive area indicated that the loss of TAOK1 significantly lowered lipid deposition in IHHs (Figure 3C, D). We also detected an increase in β-oxidation and the secretion of *de novo* synthesized TAG into the media in TAOK1-deficient IHHs (Figure 3E, F). Consistently, we found higher mitochondrial activity and content as evidenced by the enhanced staining of MitoTracker Red and Green, respectively, in IHHs transfected with *TAOK1* siRNA versus NTC siRNA (Figure 3C, D). In parallel, the silencing of TAOK1 in IHHs significantly suppressed fatty acid influx and incorporation of [^3^H]-glucose and [^3^H]-oleic acid into intracellular TAG (Figure 3G–I). Notably, the impact of TAOK1 knockdown on hepatocellular lipid metabolism was similar in IHHs cultured with or without oleate supplementation.

To investigate the metabolic effect of TAOK1 overexpression in human hepatocytes, we transfected IHHs with human *MYC*-tagged *TAOK1* expression plasmid (Supplemental Figure S5A, B, http://links.lww.com/HC9/A120). A robust increase (about 300-fold) in the *TAOK1* transcript levels was accompanied by a relatively modest rise (about 2-fold) in protein levels, which is likely explained by nonsense-mediated mRNA decay, triggered by the termination codon of the ORF positioned upstream of the most 3′ splice site in the expression plasmid.^30^ In contrast to our observations in TAOK1-deficient hepatocytes, the Bodipy-positive area was about 1.5- to 2-fold higher in IHHs transfected with *MYC-TAOK1* compared with cells transfected with empty vector (Supplemental Figure S5C, http://links.lww.com/HC9/A120 D), which was paralleled by lower mitochondrial biogenesis and reduced fatty acid oxidation (Supplemental Figure S5C, D, S6A, http://links.lww.com/HC9/A120). In contrary, *de novo* lipogenesis was activated in TAOK1-overexpressing IHHs (Supplemental Figure S6B, C, http://links.lww.com/HC9/A120). We did not detect any difference in fatty acid uptake rate in IHHs with increased TAOK1 abundance (Supplemental Figure S6D, http://links.lww.com/HC9/A120) and TAG secretion was slightly lower only in TAOK1-overexpressing cells cultured under basal conditions (Supplemental Figure S6E, http://links.lww.com/HC9/A120).

### TAOK1 regulates lipolysis and autophagic flux in human hepatocytes

**Figure 4:**
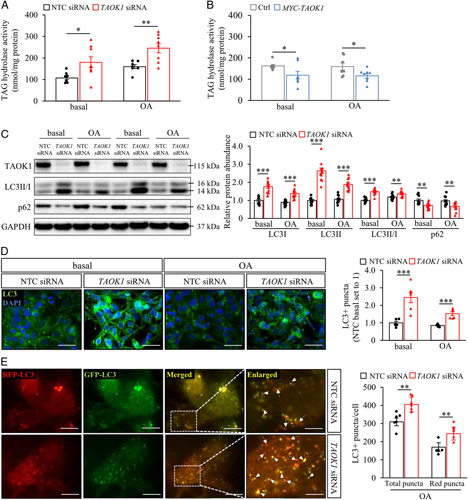
TAOK1 regulates lipolysis and autophagic flux in human hepatocytes. IHHs were transfected with *TAOK1* siRNA or NTC siRNA (A and C–E), or with *MYC*-tagged *TAOK1* expression plasmid or an empty control plasmid (B), and cultured with or without oleate supplementation as indicated. (A-B) TAG hydrolase activity was measured using [^3^H]triolein as the substrate. (C) Cell lysates were analyzed by western blot using antibodies specific for LC3, p62, or TAOK1. Protein levels were analyzed by densitometry; representative western blots are shown with GAPDH used as a loading control. (D) Representative images of cells stained with LC3 (green); nuclei stained with DAPI (blue). The scale bars represent 50 µm. Quantification of the staining. (E) Representative images of cells transfected with Tendom Sensor RFP-GFP-LC3B; merged image shows colocalization of RFP-LC3 (red) and GFP-LC3 (green) in yellow (arrows indicate autolysosomes while arrowheads indicate autophagosomes). The scale bars represent 10 µm (3 µm in the enlarged view). Quantification of the staining. Data are mean±SEM from 6 to 8 (A, B and D, E) or 12 (C) wells per group. *p<0.05, **p<0.01, ***p<0.001. Abbreviations: Ctrl, control; GAPDH, glyceraldehyde-3-phosphate dehydrogenase; NTC, nontargeting control; OA, oleic acid; RFP, red fluorescent protein; TAG, triacylglycerol; TAOK1, thousand and one kinase 1.

On the basis of the close association between TAOK1 and intrahepatocellular LDs, we next examined the hypothesis that TAOK1 impacts on lipid mobilization from the droplets locally by enhancing canonical lipolysis. In this enzymatic process, a series of lipases [adipose triglyceride lipase (ATGL), hormone-sensitive lipase (HSL), and monoglyceride lipase (MGL)] act sequentially on the surface of LDs to reduce TAG into free fatty acids, which are then either processed via β-oxidation in the mitochondria or used for the synthesis and secretion of VLDL-TAG in the ER and Golgi.^4^ Indeed, we found that lipolysis was significantly activated in IHHs transfected with *TAOK1* siRNA versus NTC siRNA (Figure 4A). Reciprocally, lipolysis was suppressed in IHHs overexpressing TAOK1 (Figure 4B).

Within the past decade, selective autophagy (also called lipophagy) has emerged as an alternative mechanism for hepatic LD consumption. Here, LDs are first engulfed by a membrane bilayer to form an autophagosome, that then fuses with a degradative lysosome to form autolysosome, in which TAGs are hydrolyzed by lysosomal acid lipase to free fatty acids to be released for mitochondrial β-oxidation.^4^ To evaluate the possible role of increased autophagy in enhanced lipid catabolism observed in TAOK1-deficient hepatocytes, we next compared the abundance of autophagic markers in IHHs transfected with *TAOK1* siRNA versus NTC siRNA. We found that the silencing of TAOK1 significantly stimulated the conversion of LC3I to LC3II and increased the number of LC3II-positive puncta (Figure 4C, D), which are the well-established markers of enhanced autophagic flux. Furthermore, we used the GFP-RFP-LC3 sensor that contains an acid-labile GFP and acid-resistant red fluorescent protein (RFP) to distinguish between autophagosome (red and green overlap resulting in yellow) and autolysosome (acidic environment resulting in red labeling only) localization of LC3.^31^ We found that both the number of yellow and red LC3 puncta representing LC3-positive autophagosomes and autolysosomes, respectively, was significantly higher in oleate-loaded TAOK1-deficient IHHs (Figure 4E). We also detected a decreased abundance of p62 protein in IHHs transfected with *TAOK1* siRNA versus NTC siRNA (Figure 4C), which is observed in the conditions of autophagic induction.^32^ Together, these results suggest that the silencing of TAOK1 stimulates autophagy in human hepatocytes.

### TAOK1 modulates hepatocellular carbohydrate metabolism

**Figure 5:**
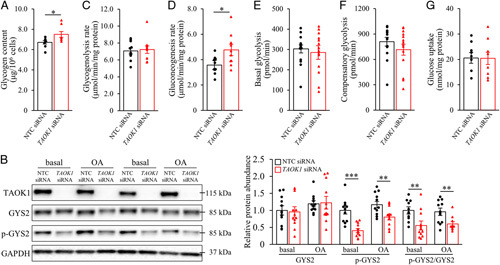
The silencing of TAOK1 has an impact on carbohydrate metabolism in human hepatocytes. IHHs were transfected with *TAOK1* siRNA or NTC siRNA and cultured only with oleate supplementation (A, C–G) or both with or without oleate supplementation as indicated (B). (A) Measurement of glycogen levels. (B) Cell lysates were analyzed by western blot using antibodies specific for GYS2, phospho-GYS2 (Ser^641^), or TAOK1. Protein levels were analyzed by densitometry; representative western blots are shown with GAPDH used as a loading control. The rate of glucose release via glycogenolysis (C) and glucose production from gluconeogenesis (D). Basal (E) and compensatory glycolysis (F) were determined under basal condition and after sequential injection of rotenone/antimycin A and 2-deoxy-d-glucose, respectively. (G) Glucose uptake was assessed in the presence of insulin. Data are mean±SEM from 8–14 wells per group. *p<0.05, **p<0.01, ***p<0.001. Abbreviations: GAPDH, glyceraldehyde-3-phosphate dehydrogenase; GYS2, glycogen synthase 2; NTC, nontargeting control; OA, oleic acid; TAOK1, thousand and one kinase 1.

In addition to lipids, glycogen serves as a major storage form of cellular energy. Interestingly, we detected a significantly higher glycogen content in oleate-treated IHHs transfected with *TAOK1* siRNA versus NTC siRNA (Figure 5A). Conceptually, increased intracellular glycogen levels in TAOK1-deficient IHHs could be caused by enhanced glycogen production or reduced glycogenolysis, or any combination of these mechanisms. To this end, we found that even if the protein abundance of the key rate-limiting enzyme in glycogen biosynthesis—glycogen synthase 2 (GYS2; active form)—was unchanged, the amount of phospho-GYS2 (Ser^641^; inactive form) was lower, in IHHs where TAOK1 was knocked down (Figure 5B). Notably, the silencing of TAOK1 in IHHs had no effect on glycogenolysis (Figure 5C).

We also observed that gluconeogenesis (quantified by measuring the glucose production rate from pyruvate and lactate) was augmented in TAOK1-deficient IHHs (Figure 5D), which may relate to the increase in β-oxidation (Figure 3E). Glycolytic rate measured by the Seahorse XF Analyzer under baseline conditions, and compensatory glycolysis assessed after the inhibition of mitochondrial oxidative phosphorylation with rotenone and antimycin A, were similar in IHHs transfected with *TAOK1* siRNA versus NTC siRNA (Figure 5E, F), and glucose uptake was unaffected (Figure 5G).

### TAOK1 regulates oxidative/ER stress in human hepatocytes

**Figure 6:**
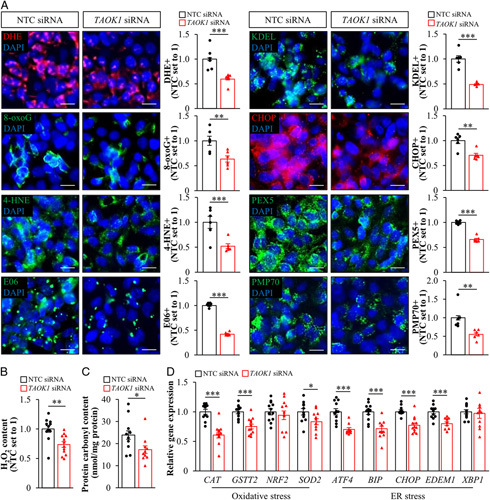
The inhibition of TAOK1 lowers oxidative and ER stress in human hepatocytes. IHHs were transfected with *TAOK1* siRNA or NTC siRNA and cultured with oleate supplementation. (A) Representative images of cells stained with DHE (red) or processed for immunofluorescence with anti-8-oxoG (green), anti-4-HNE (green), anti-E06 (green), anti-KDEL (green), anti-CHOP (red), anti-PEX5 (green), or anti-PMP70 (green) antibodies; nuclei stained with DAPI (blue). The scale bars represent 10 µm. Quantification of the staining. (B) Quantification of H_2_O_2_ content. (C) Measurement of protein carbonylation levels. (D) Relative mRNA expression of selected genes controlling oxidative and endoplasmic reticulum stress was assessed by qRT-PCR. Data are mean±SEM from 6 (A) or 10–12 (B–D) wells per group. *p<0.05, **p<0.01, ***p<0.001. Abbreviations: 4-HNE, 4-hydroxynonenal; CHOP, C/EBP-homologous protein; DHE, dihydroethidium; NTC, nontargeting control; PEX5, peroxisomal biogenesis factor 5; PMP70, peroxisomal membrane protein 70 kDa; TAOK1, thousand and one kinase 1.

Excessive lipid accumulation in hepatocytes is known to contribute to oxidative and ER stress, initiating the process of inflammation, cell death, and fibrogenesis in NASH.^33^ We therefore assessed oxidative and ER stress markers in oleate-loaded hepatocytes where TAOK1 was knocked down or overexpressed. We found that lower intrahepatocellular lipid accumulation in TAOK1-deficient IHHs was accompanied by decreased oxidative stress as evidenced by suppressed superoxide radical (O^•−^) and H_2_O_2_ content quantified by immunostaining for DHE and cellular DCFDA assay, respectively; reduced deposition of lipid peroxidation products and oxidized phospholipids measured by immunostaining for 4-HNE and E06, respectively; and diminished DNA and protein oxidation detected by immunostaining for 8-oxoG and protein carbonylation assay, respectively (Figure 6A–C). In parallel, we found that the silencing of TAOK1 in IHHs protected against ER stress as shown by reduced abundance of KDEL (an ER retrieval motif) and CHOP (an ER stress-induced apoptosis indicator) (Figure 6A, Supplemental Figure S7A, http://links.lww.com/HC9/A120). Consistently, the mRNA expression of several oxidative/ER stress markers was significantly lower in IHHs transfected with *TAOK1* siRNA versus NTC siRNA (Figure 6D).

In contrary, the overexpression of TAOK1 in IHHs resulted in exacerbated oxidative damage and ER stress as evidenced by a significant increase in the area stained with DHE, 8-oxoG, 4-HNE, E06, KDEL, and CHOP (Supplemental Figure S8, http://links.lww.com/HC9/A120).

Interestingly, we observed lower or higher peroxisomal activity, as indicated by altered levels of PEX5 (a peroxisome biogenesis marker) and PMP70 (a peroxisomal membrane marker), in IHHs where TAOK1 was silenced or overexpressed, respectively (Figure 6A, Supplemental Figure S7A, Supplemental Figure S8, http://links.lww.com/HC9/A120).

### TAOK1 may influence the susceptibility to HCC

**Figure 7:**
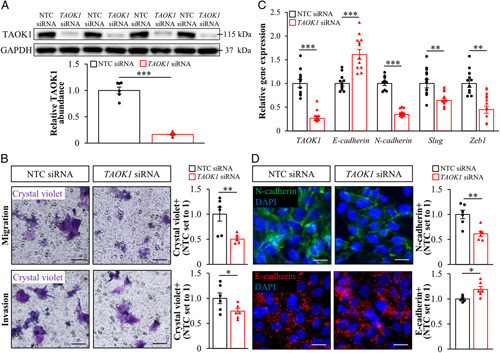
The silencing of TAOK1 inhibits mobility, invasiveness, and epithelial-mesenchymal transition in human hepatoma-derived cells. HepG2-NTCP cells were transfected with *TAOK1* siRNA or NTC siRNA and cultured with oleate supplementation. (A) TAOK1 protein abundance. Protein levels were analyzed by densitometry; representative western blots are shown with GAPDH used as a loading control. (B) Representative images of cells stained with crystal violet. The scale bars represent 20 µm. Quantification of the staining. (C) Relative mRNA expression of TAOK1 and selected genes controlling epithelial-mesenchymal transition was assessed by qRT-PCR. (D) Representative images of cells processed for immunofluorescence with anti-N-cadherin (green) or anti-E-cadherin (red) antibodies; nuclei stained with DAPI (blue). The scale bars represent 10 µm. Quantification of the staining. Data are mean±SEM from 6 (A–B and D) or 12 (C) wells per group. *p<0.05, **p<0.01, ***p<0.001. Abbreviations: GAPDH, glyceraldehyde-3-phosphate dehydrogenase; NTC, nontargeting control; TAOK1, thousand and one kinase 1

NAFLD has recently emerged as the leading cause of HCC, which is one of the most harmful malignant tumors.^3^ By analysis of the microarray GEO data sets of the 2 large cohorts of HCC subjects (n=91 for GSE102079 and n=214 for GSE14520), we found that *TAOK1* gene expression was significantly higher in HCC tumors than in adjacent nontumor liver tissue (*p*<0.0001). To further study the cell-autonomous mode of action of TAOK1 in the pathogenesis of HCC, we examined the proliferation, apoptosis, migration, and invasion, as well as the expression of epithelial-mesenchymal transition markers in TAOK1-deficient oleate-loaded HepG2-NTCP cells. We found that proliferation measured by EdU labeling assay was notably diminished in HepG2-NTCP cells where TAOK1 was knocked down and apoptosis quantified by Apopxin Green-positive cells and by caspase 3/7 activity was also slightly lowered (Supplemental Figure S9, http://links.lww.com/HC9/A120). Transwell assays revealed significant suppression of migratory and invasive capacity in HepG2-NTCP cells transfected with *TAOK1* siRNA versus NTC siRNA (Figure 7A, B). Furthermore, the silencing of TAOK1 reduced the levels of the mesenchymal marker N-cadherin and, conversely, increased the abundance of the epithelial marker E-cadherin (Figure 7C, D, Supplemental Figure S7B, http://links.lww.com/HC9/A120). Consistently, the mRNA expression of *Slug* and *Zeb1*, 2 transcription factors critical for epithelial-mesenchymal transition of cancer cells,^34^ was decreased in HepG2-NTCP cells where TAOK1 was knocked down (Figure 7C). In line with the results obtained in IHHs, we observed lower intrahepatocellular lipid storage in TAOK1-deficient HepG2-NTCP cells, which was accompanied by accelerated β-oxidation and TAG secretion, suppressed fatty acid influx and TAG synthesis, as well as diminished oxidative/ER stress (Supplemental Figure S10, http://links.lww.com/HC9/A120).

### TAOK1 disruption in human hepatocytes alters the metabolic and pro-oncogenic pathways

**Figure 8:**
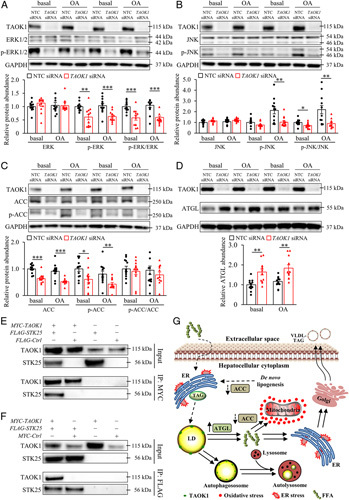
TAOK1 interacts with STK25 and affects metabolic and pro-oncogenic pathways. IHHs were transfected with *TAOK1* siRNA or NTC siRNA and cultured with or without oleate supplementation as indicated. Cell lysates were analyzed by western blot using antibodies specific for ERK or phospho-ERK (Thr^202^/Tyr^204^) (A), JNK or phospho-JNK (Thr^183^/Tyr^185^) (B), ACC or phospho-ACC (Ser^79^) (C), ATGL (D), or TAOK1. Protein levels were analyzed by densitometry; representative western blots are shown with GAPDH used as a loading control. Data are mean ± SEM from 11 to 12 wells per group. (E-F) Co-immunoprecipitation of TAOK1 and STK25 was performed from protein extracts of IHHs transfected with *MYC-TAOK1*, *FLAG-STK25, FLAG-Control* plasmid (E), and/or *MYC-Control* plasmid (F). Starting material (input), as well as protein immunoprecipitated using anti-MYC antibodies (E) or anti-FLAG antibodies (F) were analyzed by western blot using antibodies specific for TAOK1 or STK25; representative western blots are shown. (G) A working model of the function of TAOK1 in regulating hepatocellular lipotoxicity. The silencing of TAOK1 in hepatocytes inhibits LD anabolism through suppressing fatty acid uptake and TAG synthesis, stimulates LD catabolism through facilitating β-oxidation and VLDL-TAG secretion, and alleviates oxidative and ER stress. Mechanistically, the rate of canonical lipolysis and lipophagy, which both enhance lipid mobilization from the LDs for fatty acid oxidation and TAG secretion, are stimulated by TAOK1 knockdown. Furthermore, the silencing of TAOK1 decreases ACC protein abundance, which is expected to both reduce lipogenesis and augment β-oxidation, and increases canonical ATGL lipase levels. *p<0.05, **p<0.01, ***p<0.001. Abbreviations: ACC, acetyl-CoA carboxylase; ATGL, adipose triglyceride lipase; Ctrl, control; ER, endoplasmic reticulum; FFA, free fatty acid; GAPDH, glyceraldehyde-3-phosphate dehydrogenase; IP, immunoprecipitated material; JNK, JUN N-terminal kinase; LD, lipid droplet; NTC, nontargeting control; OA, oleic acid; TAG, triacylglycerol; TAOK1, thousand and one kinase 1.

To explore the mechanisms by which TAOK1 deficiency suppresses lipotoxicity and metastatic capacity in human hepatocytes, we first monitored the phosphorylation of MAPKs extracellular signal-regulated kinase (ERK) and JNK, which are activated in human HCC.^35^ We observed significantly reduced phosphorylation of ERK1/2 (Thr^202^/Tyr^204^) and JNK1/2 (Thr^183^/Tyr^185^) in IHHs transfected with *TAOK1* siRNA versus NTC siRNA (Figure 8A, B). Interestingly, we also found that the silencing of TAOK1 lowered the protein abundance of acetyl-CoA carboxylase (ACC)—a key regulator of lipid metabolism that suppresses β-oxidation and stimulates TAG synthesis 36—without any change in the ratio of phospho-ACC (Ser^79^; inactive form)/ACC (active form) (Figure 8C). Furthermore, the abundance of ATGL, the first lipase in the TAG hydrolysis pathway, was significantly increased in TAOK1-deficient IHHs [Figure 8D; HSL/phospho-HSL (Ser^660^) remained below the level of quantification]. Recently, TAOK proteins were reported to activate yes-associated protein (YAP) signaling by means of LATS1/2 phosphorylation in HEK293 cells.^20^,^37^,^38^ Here, we found no significant difference in the activation of LATS1 or YAP in IHHs transfected with *TAOK1* siRNA versus NTC siRNA (Supplemental Figure S11, http://links.lww.com/HC9/A120). Notably, the total protein level of AKT was reduced, whereas the amount of phospho-AKT (Ser^473^) was elevated, in TAOK1-deficient IHHs (Supplemental Figure S12, http://links.lww.com/HC9/A120). Remarkably, changes caused by TAOK1 knockdown were largely similar in hepatocytes cultured under basal conditions or treated with oleic acid.

Our previous studies by screening a genome-wide yeast 2-hybrid (Y2H) cDNA library derived from primary human hepatocytes, identified STE20-type kinase TAOK3 as a binding partner for STK25^16^—an LD-coating protein controlling both liver lipid synthesis and utilization.^7^–^9^,^11^,^14^,^15^,^39^ Importantly, the prey fragment of TAOK3 that interacts with STK25 (amino acids 1 to 447; harbors the N-terminal kinase domain and serine-rich domain of unknown function^16^) is highly conserved compared with TAOK1 (75% similarity in amino acid sequence). To test the hypothesis that TAOK1 also binds to STK25, we transfected IHHs with plasmids encoding *MYC-TAOK1* and *FLAG-STK25*. Using anti-MYC and anti-FLAG immunoprecipitations, we were able to confirm a direct interaction between TAOK1 and STK25 proteins (Figure 8E, F). Of note, the silencing of TAOK1 in IHHs did not result in any alteration of STK25 protein abundance (Supplemental Figure S13, http://links.lww.com/HC9/A120).

### TAOK1 has no impact on the lipotoxicity of liver nonparenchymal cells

Our results demonstrating an abundant expression of TAOK1 in liver macrophages and HSCs prompted us to investigate a potential role of TAOK1 in the regulation of lipotoxicity in these cell types. We found no changes in lipid deposition or H_2_O_2_ content in THP-1-derived macrophages or LX-2 human HSC line transfected with *TAOK1* siRNA versus NTC siRNA (Supplemental Figure S14A-B, S15A-B, http://links.lww.com/HC9/A120). Consistently, the protein abundance of NADPH oxidase 2, inducible nitric oxide synthase, and TNFα—the key mediators for macrophage-associated proinflammatory state in the liver^40^,^41^—remained unaltered in TAOK1-deficient macrophages (Supplemental Figure S14C, http://links.lww.com/HC9/A120). Furthermore, the silencing of TAOK1 had no impact on the protein abundance of α smooth muscle actin (a marker for activated HSCs), TNFα (a proinflammatory marker), or TGFβ (a profibrotic mediator) in LX-2 cells (Supplemental Figure S15C, http://links.lww.com/HC9/A120).

## DISCUSSION

STE20-type kinase TAOK1 has been identified as a component of hepatocellular LD proteome,^10^,^15^ suggesting a potential role in regulating liver steatosis and NAFLD development. In this study, we sought to investigate the association between hepatic *TAOK1* expression and NAFLD severity and to decipher its mechanism of action in human hepatocytes. We observed that *TAOK1* mRNA expression in human liver biopsies was positively correlated with the key hallmarks of NAFLD (ie, hepatic steatosis, inflammation, and ballooning) and TAOK1 protein abundance was increased in livers from high-fat diet-fed mice compared with lean controls. We also found that the *in vitro* knockdown of TAOK1 protected human hepatocytes against excessive lipid storage, as well as oxidative and ER stress, and the opposite changes were detected in TAOK1-overexpressing hepatocytes.

Importantly, we show that the silencing of TAOK1 reprogrammed cellular metabolism by stimulating lipid catabolism (mitochondrial β-oxidation and TAG efflux) and inhibiting lipid anabolism (fatty acid influx and lipogenesis), collectively lowering ectopic fat storage within intrahepatocellular LDs (Figure 8G). Consistently, both the rate of canonical lipolysis and lipophagy, facilitating lipid mobilization from the LDs for β-oxidation and secretion, were significantly increased in TAOK1-deficient hepatocytes (Figure 8G). Remarkably, the alterations in lipid metabolism caused by the silencing of TAOK1 were largely similar in hepatocytes cultured with or without oleate supplementation. In parallel with the reduced fat accumulation, we observed markedly lowered incidences of oxidative/ER stress in hepatocytes where TAOK1 was knocked down. This finding is interesting in light of recent evidence demonstrating that oxidative/ER stress are key factors, which trigger NAFLD progression from simple steatosis toward NASH, as well as further aggravation to HCC.^1^ However, we surmise that, at this juncture, we cannot delineate whether the alterations in oxidative and ER stress in TAOK1-deficient hepatocytes were secondary to the reduction in cellular lipid accumulation or were mediated by a different pathway controlled by TAOK1.

Mechanistically, we found that the silencing of TAOK1 significantly suppressed the abundance of ACC protein in human hepatocytes. This provides a plausible mechanism underlying the protection against ectopic fat storage observed in TAOK1-deficient hepatocytes since the enzymatic product of ACC, malonyl-CoA, is an intermediate of lipogenesis and also represses β-oxidation by means of the inhibition of the main mitochondrial fatty acid importer carnitine palmitoyltransferase 1 (CPT1).^36^ Of note, 2 liver-directed small-molecule ACC antagonists (GS-0976 and PF-0522134) have recently demonstrated efficacy in clinical phase II trials in patients diagnosed with NAFLD or NASH,^42^–^45^ by decreasing hepatic steatosis and lowering serum markers of liver injury/fibrosis, which highlights the potential of ACC as a drug discovery target in metabolic liver disease. Furthermore, we detected elevated levels of ATGL protein in hepatocytes where TAOK1 was silenced, which is expected to impact on the increased lipid utilization by enhancing canonical lipolysis rate.^46^–^48^ In parallel, we found that the knockdown of TAOK1 in hepatocytes suppressed phosphorylation of ERK and JNK, which are critical signaling components stimulating proliferation, migration, and invasion of NASH-driven HCC.^35^ Consistently, we observed lower proliferative, migratory, and invasive capacity, as well as epithelial-mesenchymal transition in TAOK1-deficient hepatoma-derived cells. Interestingly, the inhibition of hepatic JNK activity has also been shown to increase fatty acid oxidation and decrease lipogenesis, thus alleviating liver steatosis.^49^ Hence, reduced JNK signaling in TAOK1-deficient hepatocytes may have contributed to the observed alterations in lipid metabolism; however, this possibility has not been further investigated in the current study.

Similarly to TAOK1, a closely related STE20 kinase TAOK3 (also known as MAP3K18, JIK, or DPK) has been demonstrated to associate with intrahepatocellular LDs, promoting ectopic fat storage and aggravating oxidative/ER stress.^16^ Interestingly, both TAOK1 and TAOK3 interact with another LD-coating STE20-type kinase—STK25 (Figure 8E, F,^16^). Our recent studies in cultured human hepatocytes and mouse models have revealed that STK25 deficiency protects against liver steatosis by shifting the metabolic balance from lipid anabolism towards lipid catabolism.^7^–^9^,^11^,^14^,^15^ This raises the possibility that TAOK1/TAOK3 and STK25 function in the same signaling pathway and that interaction with STK25 may play a part in the molecular mechanism of action of TAOK1/TAOK3 in the regulation of hepatocellular lipotoxic milieu. Notably, in addition to TAOK1 and TAOK3, the human GCKVIII subfamily of STE20-type kinases also includes TAOK2 (also known as MAP3K17 or PSK1), which shares about 90% amino acid identity with TAOK1/TAOK3 in the N-terminal protein kinase domain and displays about 60%–70% similarity in the central serine-rich and C-terminal regulatory domains. To date, it is not known whether the subcellular localization and function of TAOK2 in hepatocytes are similar or different compared with TAOK1 and TAOK3.

We found that, in addition to hepatocytes, TAOK1 was abundantly expressed in liver nonparenchymal cells including HSCs and macrophages. This is interesting in light of recent evidence demonstrating that macrophages and HSCs are susceptible to lipotoxic damage characterized by excessive fat storage and oxidative stress, and causally contribute to NASH initiation and progression by stimulating inflammation and fibrogenesis.^40^,^50^ Notably, in this study, we did not find any evidence that the silencing of TAOK1 would reduce lipid storage or oxidative stress in liver nonparenchymal cells.

The present study does have some limitations. Primarily, all the *in vitro* experiments in this report were performed using immortalized human cell lines, which may not be representative of *in vivo* conditions. To this end, further investigations using mouse models and human primary cells are warranted. Importantly, although this report provides mechanistic insight into the regulatory role of TAOK1 in the control of liver lipotoxicity, we have not yet fully characterized the hepatocellular mode of action of TAOK1, including its upstream activators or downstream substrates, which will be the focus of our future studies.

In conclusion, our data shows for the first time that LD-binding STE20-type kinase TAOK1 modulates hepatocellular lipid homeostasis and, through control of the lipid channeling between anabolic and catabolic pathways, its deficiency breaks the vicious cycle of excessive lipid storage and oxidative/ER stress within hepatocytes.

## Supplementary Material
