# Supplementary material for: Silencing of STE20-type kinase TAOK1 confers protection against hepatocellular lipotoxicity through metabolic rewiring
Source: Hepatol Commun. 2023 Mar 17;7(4):e0037. doi: 10.1097/HC9.0000000000000037 (PMC10027040; doi:10.1097/HC9.0000000000000037)
Supplement: Supplementary file 1 [file hc9-7-e0037-s001.pdf]

Supplementary Figure S1

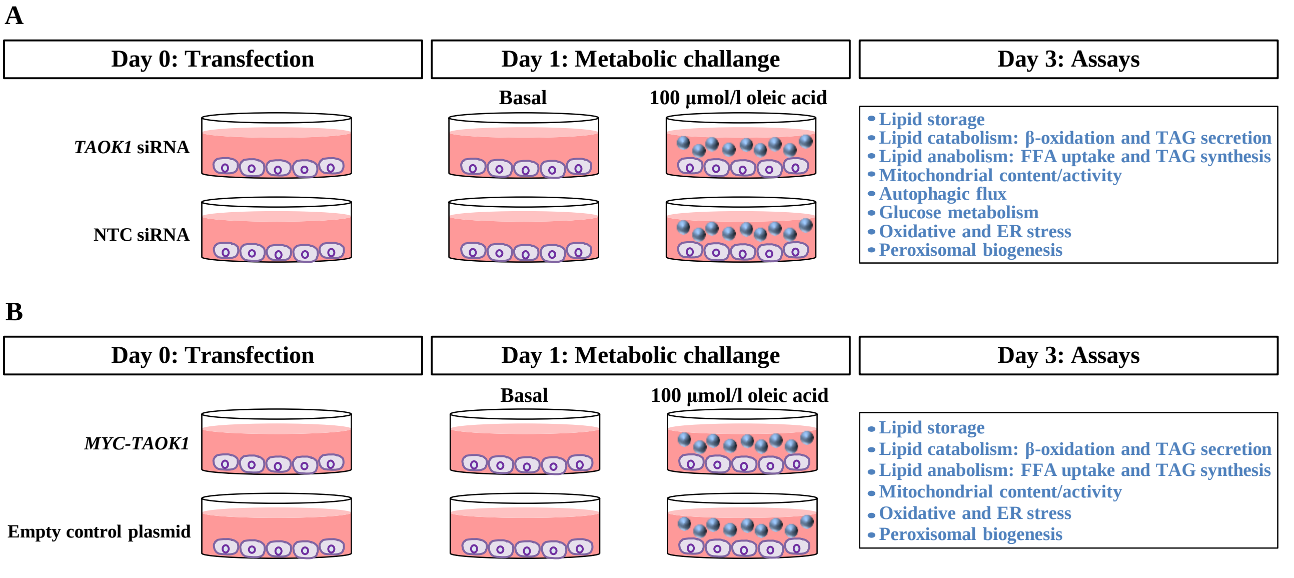

Fig. S1. Schematic illustration of the study design for *TAOK1* siRNA (A) and overexpression (B) experiments. ER, endoplasmic reticulum; FFA, free fatty acid; TAG, triacylglycerol

Supplementary Figure S2

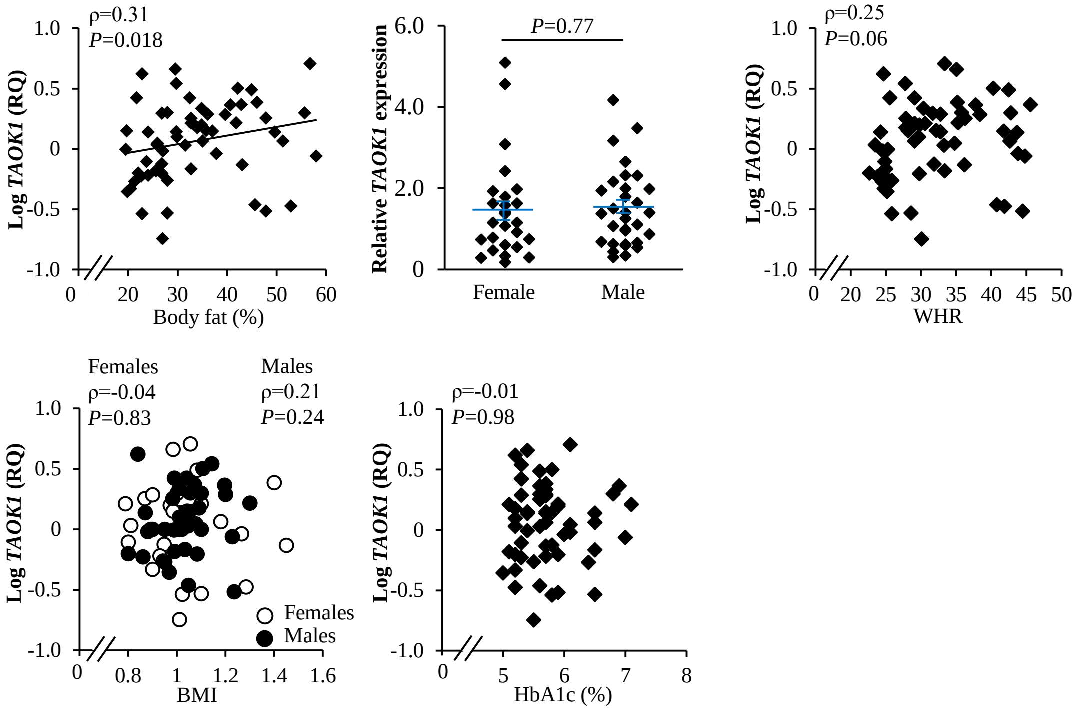

Fig. S2. The hepatic expression of *TAOK1* mRNA is positively correlated with body fat but not with gender, WHR, BMI, or whole blood HbA1c values of the subjects. *TAOK1* mRNA expression was quantified in liver biopsies by qRT-PCR. RQ, relative quantification

Supplementary Figure S3

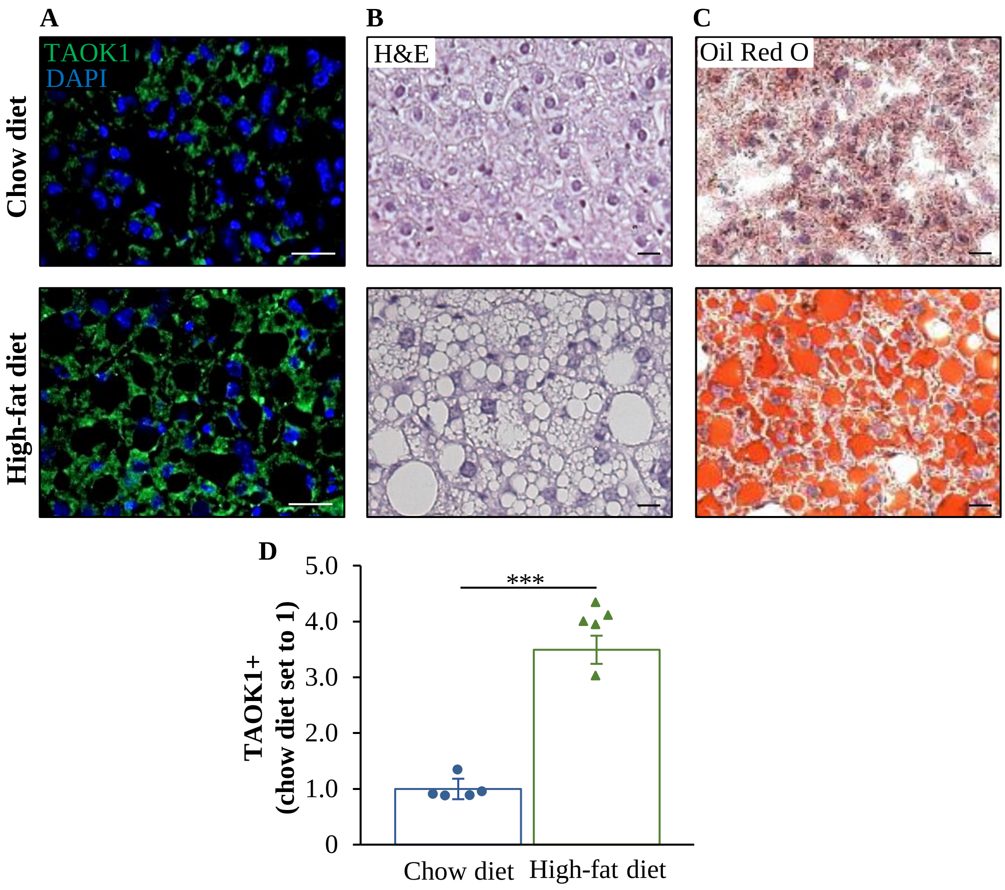

Fig. S3. Expression level of TAOK1 is increased in the livers from mice fed a high-fat diet compared with age-matched chow-fed controls. (A-C) Representative images of liver sections processed for immunofluorescence with anti-TAOK1 (green) antibodies; nuclei stained with DAPI (blue) (A) or stained with H&E (B) or Oil Red O (C). The scale bars represent 20 µm. (D) Quantification of the staining of TAOK1. Data are mean ± SEM from 5 mice per group. ****P*<0.001

Supplementary Figure S4

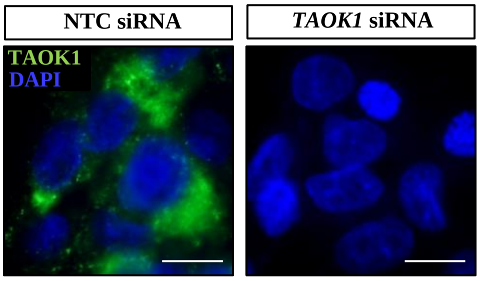

Fig. S4. Immunostaining for TAOK1 is substantially reduced in human hepatocytes transfected with *TAOK1* siRNA. IHHs were transfected with *TAOK1* siRNA or NTC siRNA and cultured with oleate supplementation. Representative images of cells processed for immunofluorescence with anti-TAOK1 (green) antibodies; nuclei stained with DAPI (blue). The scale bars represent 10 µm.

Supplementary Figure S5

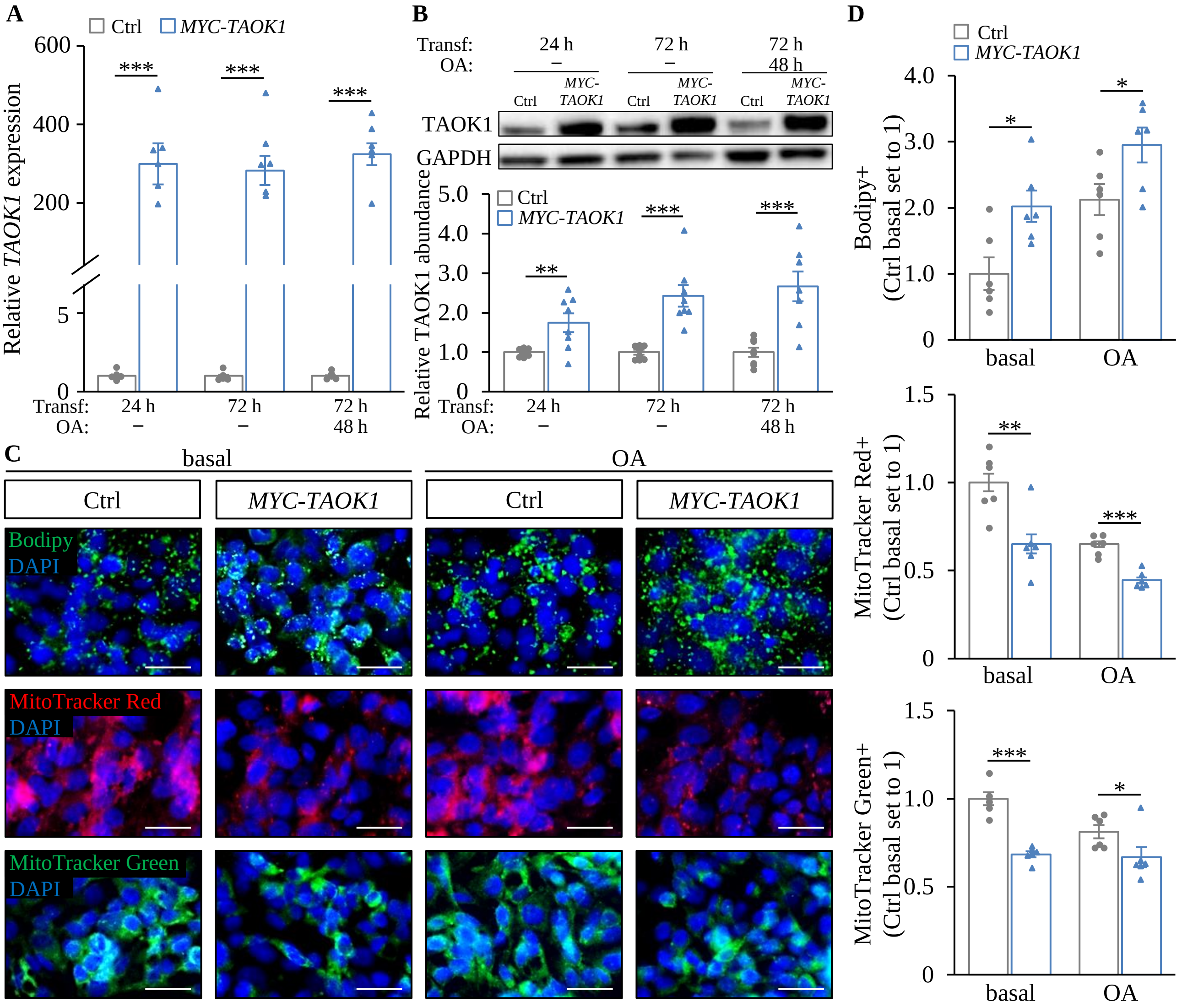

Fig. S5. Overexpression of TAOK1 aggravates lipid accumulation and mitochondrial dysfunction in human hepatocytes. IHHs were transfected with *MYC*-tagged *TAOK1* expression plasmid or an empty control plasmid, and cultured with or without oleate supplementation as indicated. (A-B) TAOK1 mRNA (A) and protein (B) abundance was assessed by qRT-PCR and Western blot, respectively. In (B), protein levels were analyzed by densitometry; representative Western blots are shown with GAPDH used as a loading control. (C-D) Representative images of cells stained with Bodipy (green), MitoTracker Red (red), or MitoTracker Green (green); nuclei stained with DAPI (blue) (C). The scale bars represent 20 µm. Quantification of the staining (D). Data are mean ± SEM from 6-8 wells per group. Ctrl, control; OA, oleic acid; Transf, transfection. **P*<0.05, ***P*<0.01, ****P*<0.001

Supplementary Figure S6

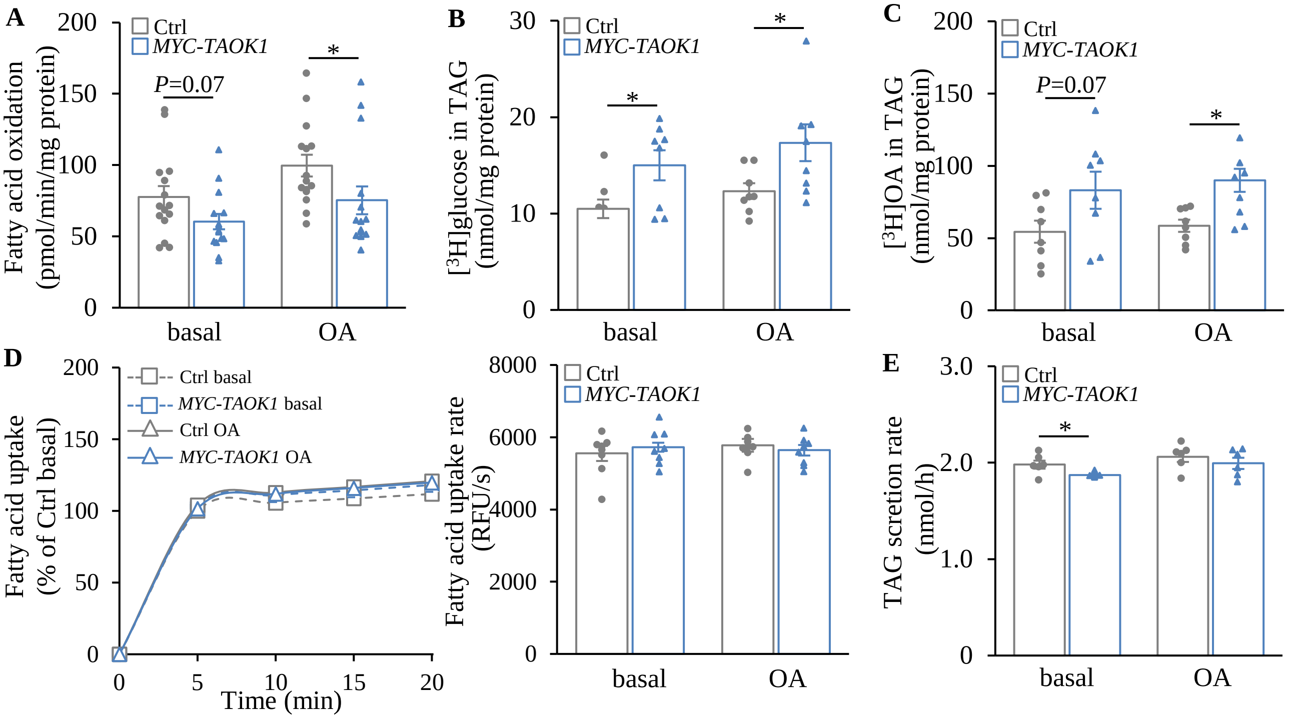

Fig. S6. Overexpression of TAOK1 reduces lipid catabolism and accelerates TAG synthesis in human hepatocytes. IHHs were transfected with *MYC*-tagged *TAOK1* expression plasmid or an empty control plasmid, and cultured with or without oleate supplementation as indicated. (A) Oxidation of radiolabeled palmitate. (B-C) TAG synthesis from [^{3}H]-labeled glucose (B) and [^{3}H]-labeled oleic acid (C). (D) Fatty acid uptake rate. (E) Secretion of [^{3}H]TAG into the media. Data are mean ± SEM from 6-8 (B-E) or 15 (A) wells per group. Ctrl, control; OA, oleic acid; Transf, transfection. *P<0.05

Supplementary Figure S7

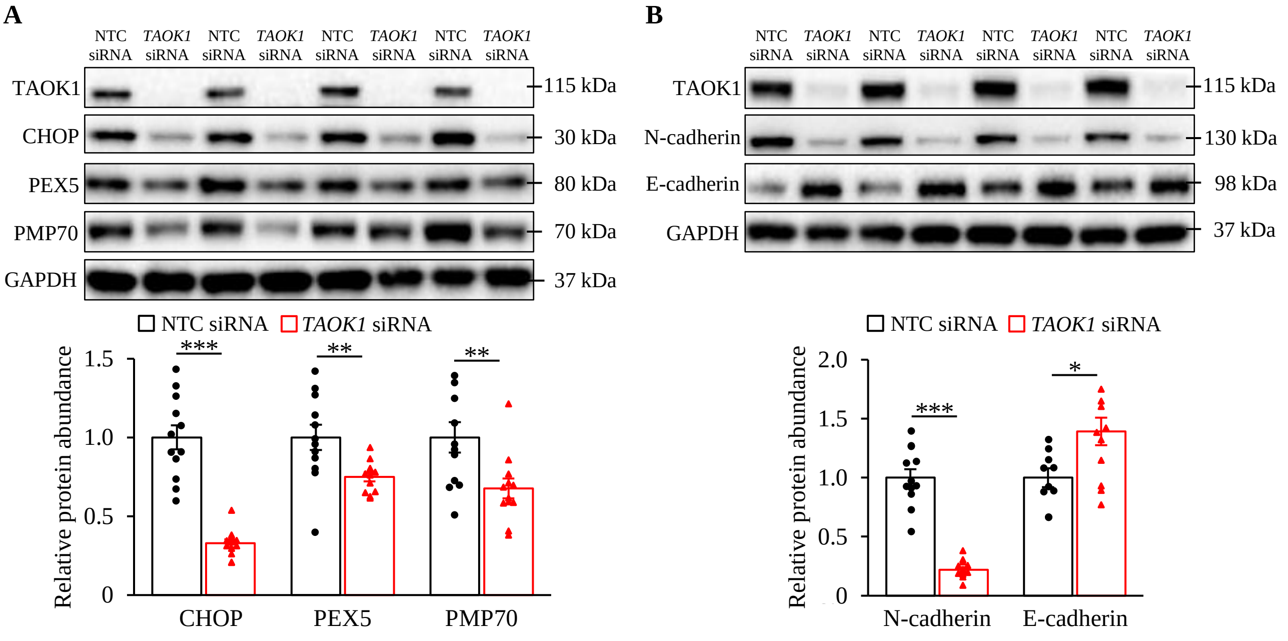

Fig. S7. The inhibition of TAOK1 lowers ER stress, peroxisomal activity, and EMT in human hepatocytes. IHHs (A) or HepG2-NTCP cells (B) were transfected with *TAOK1* siRNA or NTC siRNA and cultured with oleate supplementation. Cell lysates were analyzed by Western blot using antibodies specific for CHOP, PEX5, PMP70, N-cadherin, E-cadherin, or TAOK1. Protein levels were analyzed by densitometry; representative Western blots are shown with GAPDH used as a loading control. Data are mean ± SEM from 10-12 wells per group. *P<0.05, **P<0.01, ***P<0.001

Supplementary Figure S8

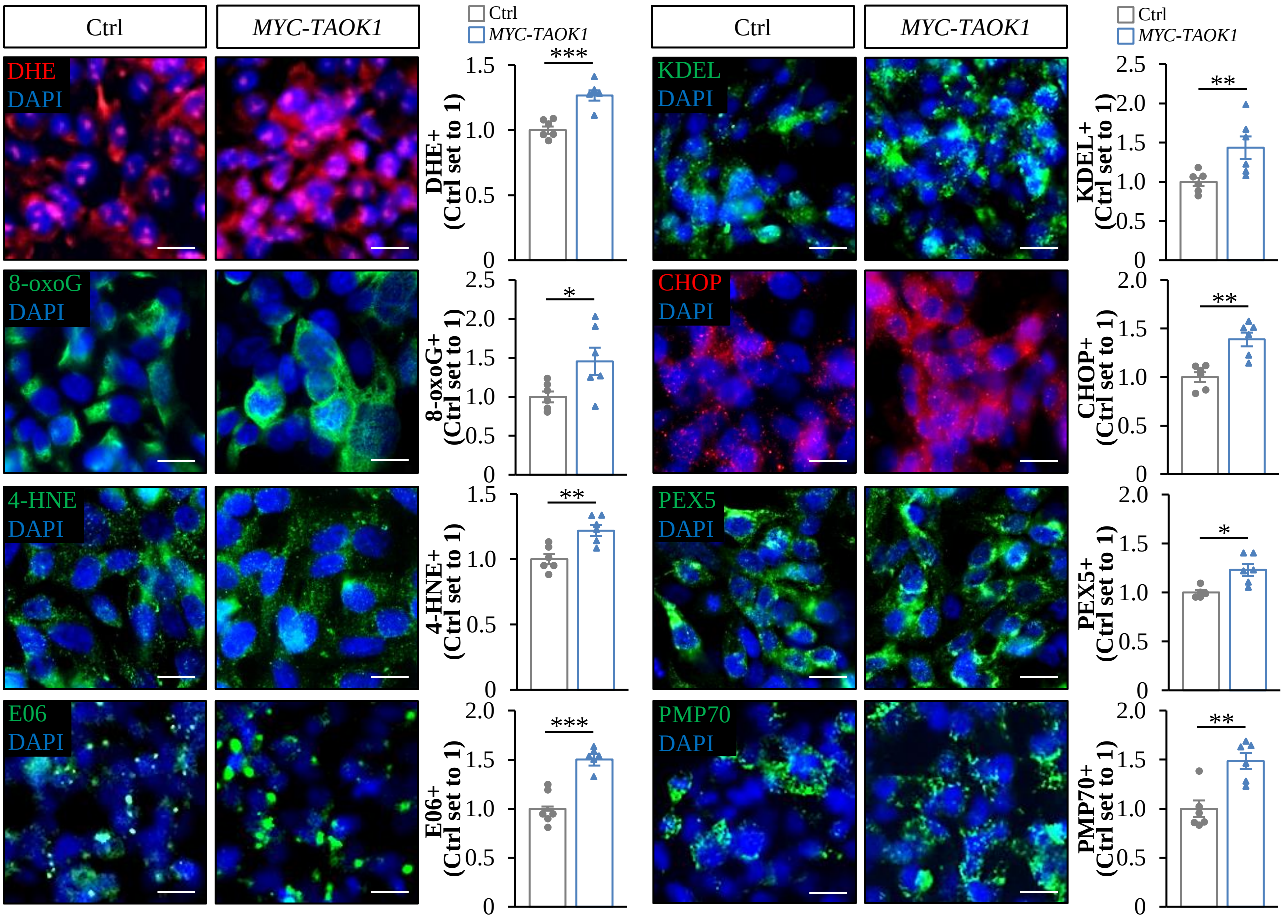

Fig. S8. Overexpression of TAOK1 exacerbates oxidative and ER stress in human hepatocytes. IHHs were transfected with *MYC*-tagged *TAOK1* expression plasmid or an empty control plasmid, and cultured with oleate supplementation. Representative images of cells stained with DHE (red) or processed for immunofluorescence with anti-8-oxoG (green), anti-4-HNE (green), anti-E06 (green), anti-KDEL (green), anti-CHOP (red), anti-PEX5 (green), or anti-PMP70 (green) antibodies; nuclei stained with DAPI (blue). The scale bars represent 10 µm. Quantification of the staining. Data are mean ± SEM from 6 wells per group. Ctrl, control. *P<0.05, **P<0.01, ***P<0.001

Supplementary Figure S9

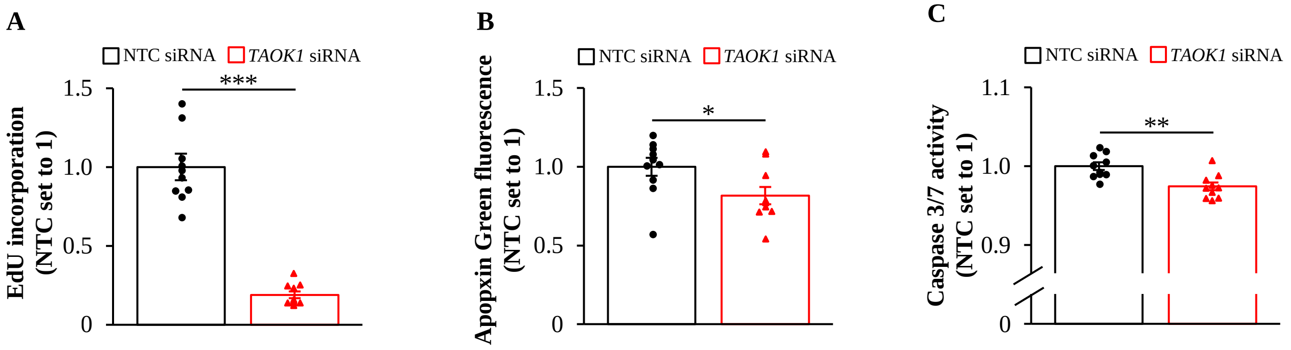

Fig. S9. The silencing of TAOK1 decreases proliferation as well as apoptosis in human hepatoma-derived cells. HepG2-NTCP cells were transfected with *TAOK1* siRNA or NTC siRNA and cultured with oleate supplementation. (A) Proliferation level assessed by measuring the DNA synthesis (Ex/Em = 568/585 nm). (B-C) Apoptosis monitored by staining with Apopxin Green (Ex/Em = 490/525 nm, B) and by analyzing caspase 3/7 activity by luminescent assay (C). Data are mean ± SEM from 10 wells per group. *P<0.05, **P<0.01, ***P<0.001

Supplementary Figure S10

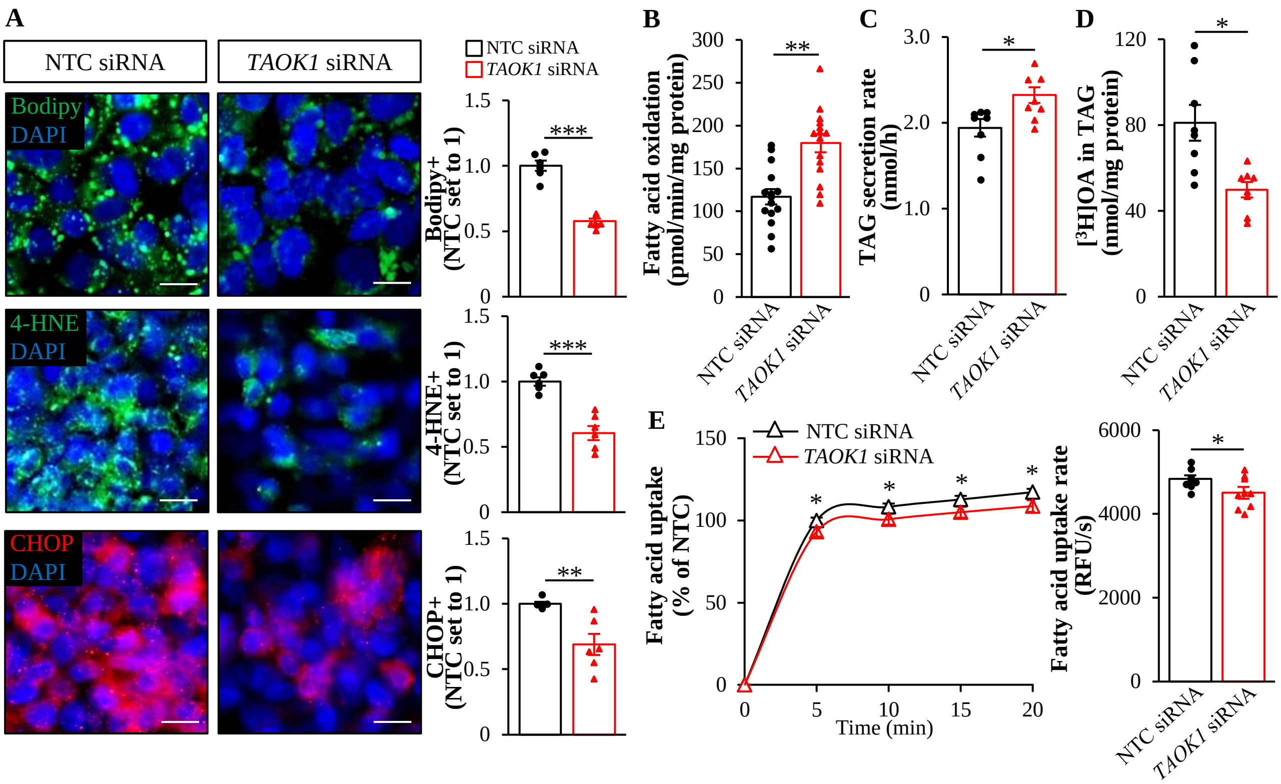

Fig. S10. The silencing of TAOK1 decreases lipid accumulation as well as oxidative/ER stress in human hepatoma-derived cells. HepG2-NTCP cells were transfected with *TAOK1* siRNA or NTC siRNA and cultured with oleate supplementation. (A) Representative images of cells stained with Bodipy (green) or processed for immunofluorescence with anti-4-HNE (green) or anti-CHOP (red) antibodies; nuclei stained with DAPI (blue). The scale bars represent 10 µm. Quantification of the staining. (B) Oxidation of radiolabeled palmitate. (C) Secretion of [^{3}H]TAG into the media. (D) TAG synthesis from [^{3}H]-labeled oleic acid. (E) Fatty acid uptake rate. Data are mean ± SEM from 6-8 (A and C-E) or 15 (B) wells per group. **P*<0.05, ***P*<0.01, ****P*<0.001

Supplementary Figure S11

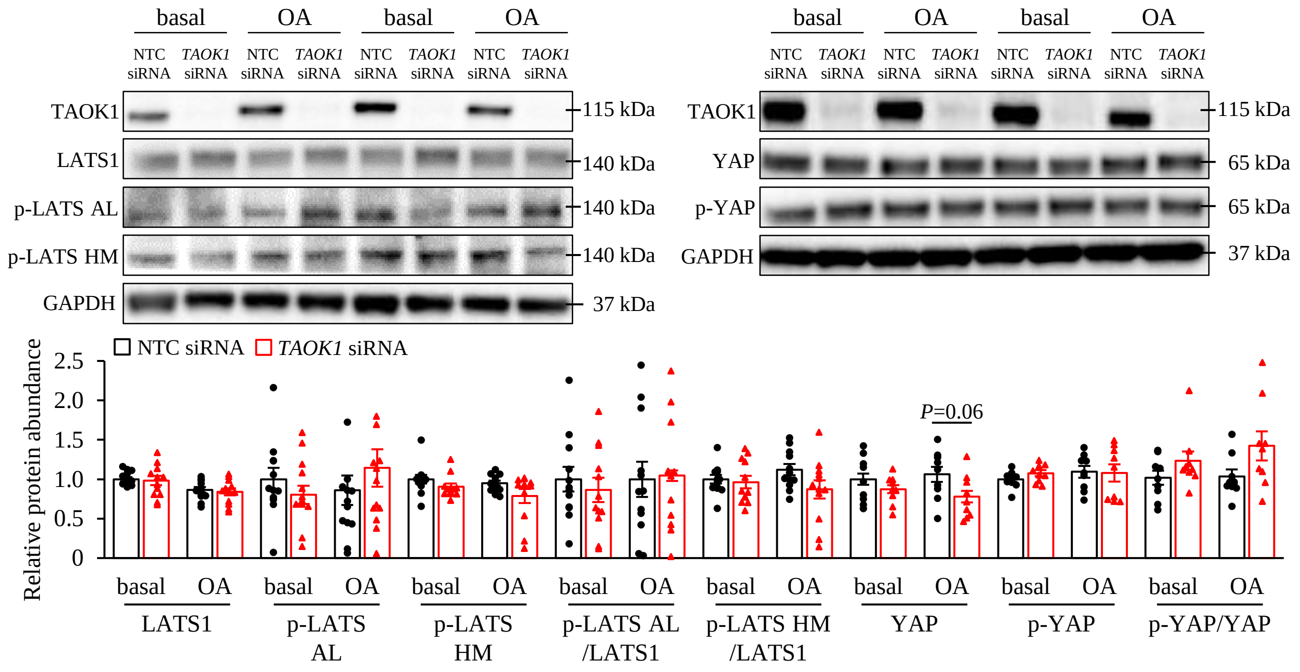

Fig. S11. LATS1 and YAP signaling is unaffected in TAOK1-deficient human hepatocytes. IHHs were transfected with *TAOK1* siRNA or NTC siRNA and cultured with or without oleate supplementation as indicated. Cell lysates were analyzed by Western blot using antibodies specific for LATS1, phospho-LATS1 AL (Ser^{909}), phospho-LATS HM (Thr^{1079}), YAP, phospho-YAP (Ser^{127}), or TAOK1. Protein levels were analyzed by densitometry; representative Western blots are shown with GAPDH used as a loading control. Data are mean ± SEM from 9-12 wells per group. OA, oleic acid

Supplementary Figure S12

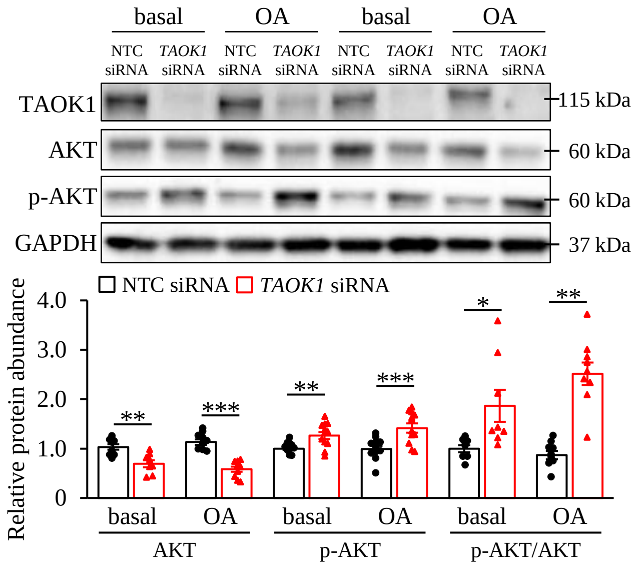

Fig. S12. AKT signaling is altered in TAOK1-deficient human hepatocytes. IHHs were transfected with *TAOK1* siRNA or NTC siRNA and cultured with or without oleate supplementation as indicated. Cell lysates were analyzed by Western blot using antibodies specific for AKT, phospho-AKT (Ser^{473}), or TAOK1. Protein levels were analyzed by densitometry; representative Western blots are shown with GAPDH used as a loading control. Data are mean ± SEM from 11-12 wells per group. OA, oleic acid. *P<0.05, **P<0.01, ***P<0.001

Supplementary Figure S13

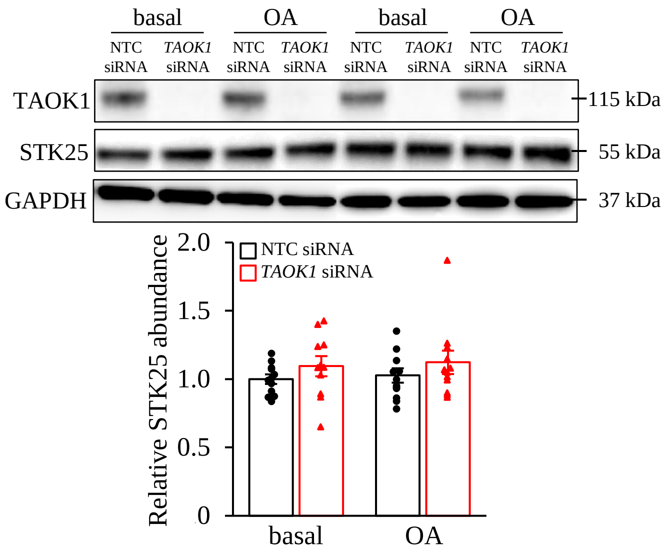

Fig. S13. The silencing of TAOK1 does not affect protein abundance of STK25 in human hepatocytes. IHHs were transfected with *TAOK1* siRNA or NTC siRNA and cultured with or without oleate supplementation as indicated. Cell lysates were analyzed by Western blot using antibodies specific for TAOK1 or STK25. Protein levels were analyzed by densitometry; representative Western blots are shown with GAPDH used as a loading control. Data are mean ± SEM from 12 wells per group. OA, oleic acid

Supplementary Figure S14

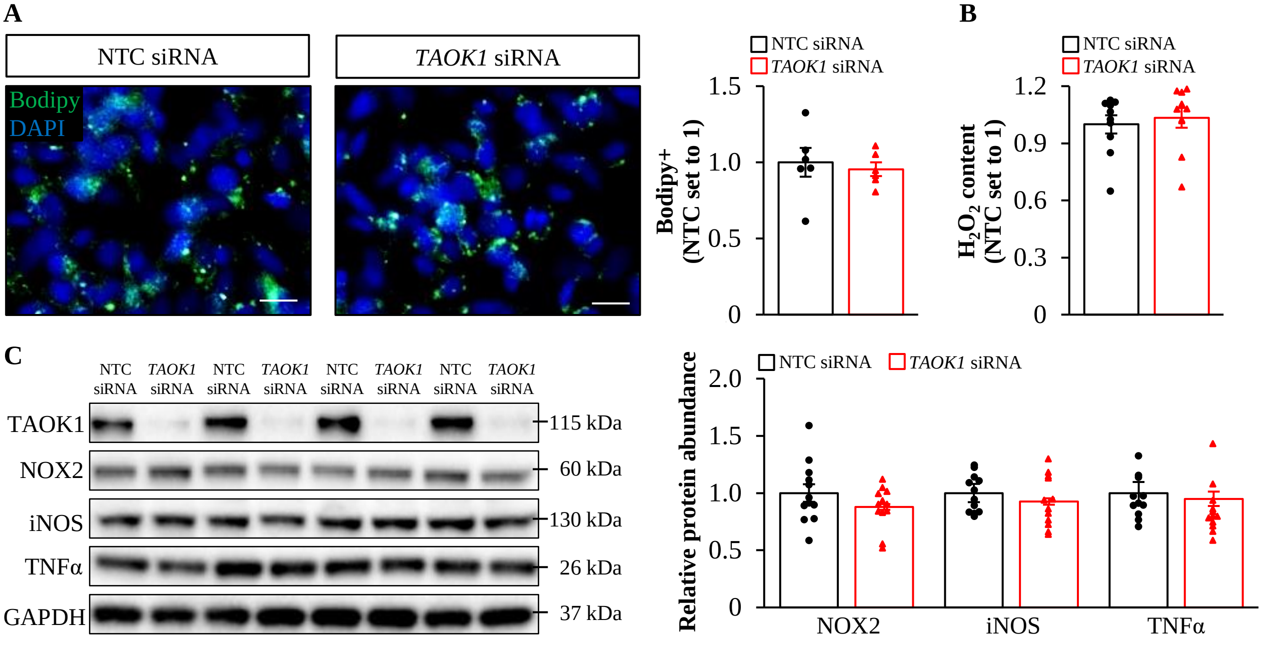

Fig. S14. The silencing of TAOK1 has no impact on the lipotoxicity of macrophages. THP-1-derived macrophages were transfected with *TAOK1* siRNA or NTC siRNA and cultured with oleate supplementation. (A) Representative images of cells stained with Bodipy (green); nuclei stained with DAPI (blue). The scale bars represent 10 µm. Quantification of the staining. (B) Quantification of H_2O_2 content. (C) Cell lysates were analyzed by Western blot using antibodies specific for NOX2, iNOS, TNFα, or TAOK1. Protein levels were analyzed by densitometry; representative Western blots are shown with GAPDH used as a loading control. Data are mean ± SEM from 6 (A) or 10-12 (B-C) wells per group.

Supplementary Figure S15

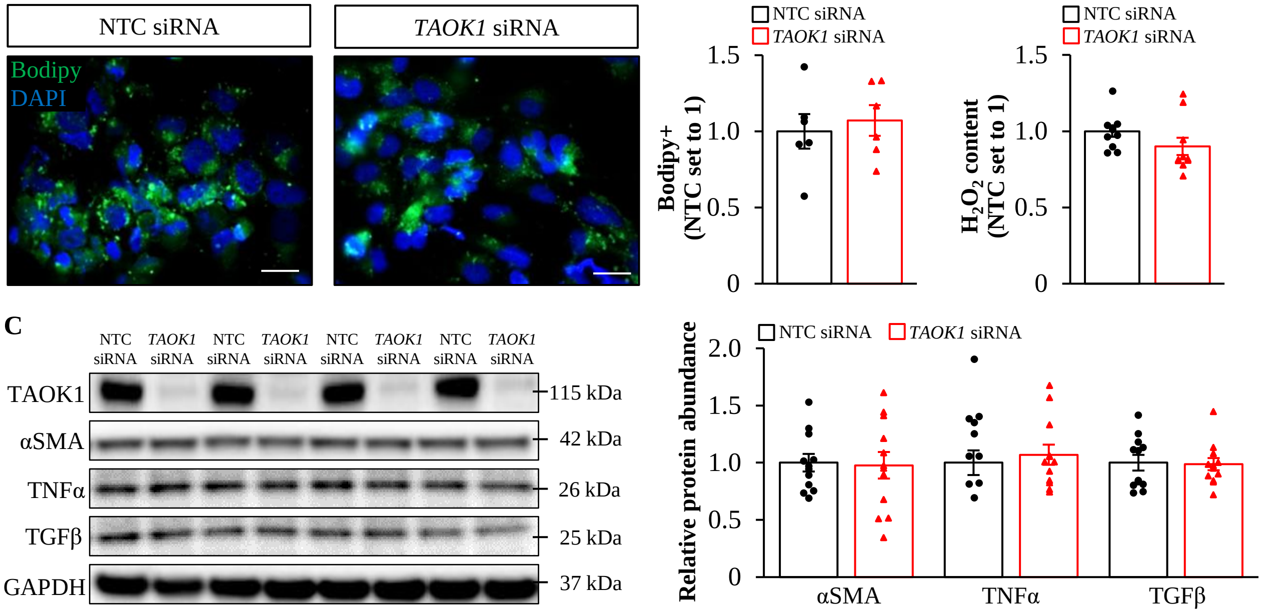

Fig. S15. The silencing of TAOK1 has no impact on the lipotoxicity of HSCs. LX-2 cells were transfected with *TAOK1* siRNA or NTC siRNA and cultured with oleate supplementation. (A) Representative images of cells stained with Bodipy (green); nuclei stained with DAPI (blue). The scale bars represent 10 µm. Quantification of the staining. (B) Quantification of H_2O_2 content. (C) Cell lysates were analyzed by Western blot using antibodies specific for αSMA, TNFα, TGFβ, or TAOK1. Protein levels were analyzed by densitometry; representative Western blots are shown with GAPDH used as a loading control. Data are mean ± SEM from 6 (A) or 10-12 (B-C) wells per group.
