# Supplementary material for: Silencing of STE20-type kinase TAOK1 confers protection against hepatocellular lipotoxicity through metabolic rewiring
Source: Hepatol Commun. 2023 Mar 17;7(4):e0037. doi: 10.1097/HC9.0000000000000037 (PMC10027040; doi:10.1097/HC9.0000000000000037)
Supplement: Supplementary file 2 [file hc9-7-e0037-s002.docx]

**Supplementary Table 1.** List of antibodies used for Western blot and immunofluorescence analysis

| **Type** | **Antibody name and catalogue number** | **Working dilution** | **Company** |  |
| --- | --- | --- | --- | --- |
| Primary | anti-TAOK1 (26250-1-AP) | 1:750 | Proteintech (Chicago, IL) | |
| antibody | anti-ADRP (ab181452) | 1:500 | Abcam (Cambridge, UK) | |
|  | anti-GFAP (13-0300) | 1:500 | Invitrogen (Waltman, MA) | |
|  | anti-F4/80 (MCA497GA) | 1:250 | Bio-Rad (Hercules, CA) | |
|  | anti-Actin (#A2920) | 1:500 | Santa Cruz Biotechnology (Santa Cruz, CA) | |
|  | anti-GAPDH (sc-47724) | 1:1000 | Santa Cruz Biotechnology | |
|  | anti-LC3 (#2775) | 1:1000 | Cell Signaling Technology (Boston, MA) | |
|  | anti-p62 (#5114) | 1:1000 | Cell Signaling Technology | |
|  | anti-GYS2 (#3886) | 1:1000 | Cell Signaling Technology | |
|  | anti-p-GYS2 (#3891) | 1:1000 | Cell Signaling Technology | |
|  | anti-8-oxoG (ab62623)  anti-4-HNE (ab46545) | 1:500  1:500 | Abcam  Abcam | |
|  | anti-E06 (330001S) | 1:100 | Avanti Polar Lipids, Inc. (Alabaster, AL) | |
|  | anti-CHOP (sc-7351) | 1:100 | Santa Cruz Biotechnology | |
|  | anti-KDEL (ab176333)  anti-CHOP (MA1-250)  anti-PEX5 (PA5-58716)  anti-PMP70 (PA1-650) | 1:500  1:200  1:500  1:500 | Abcam  Invitrogen  Invitrogen  Invitrogen | |
|  | anti-N-cadherin (33-3900) | 1:500 | Invitrogen | |
|  | anti-E-cadherin (14-3249-82) | 1:200 | Invitrogen | |
|  | anti-ERK1/2 (#9102)  anti-p-ERK1/2 (#9101) | 1:1000  1:1000 | Cell Signaling Technology  Cell Signaling Technology | |
|  | anti-JNK1/2 (#9252) | 1:1000 | Cell Signaling Technology | |
|  | anti-p-JNK1/2 (#4668) | 1:1000 | Cell Signaling Technology | |
|  | anti-ACC (#3662)  anti-p-ACC (#3661) | 1:1000  1:1000 | Cell Signaling Technology  Cell Signaling Technology | |
|  | anti-ATGL (sc-365278) | 1:500 | Santa Cruz Biotechnology | |
|  | anti-HSL (#4107) | 1:500 | Cell Signaling Technology | |
|  | anti-p-HSL (#45804) | 1:500 | Cell Signaling Technology | |
|  | anti-AKT (#9272) | 1:1000 | Cell Signaling Technology | |
|  | anti-p-AKT (#9271)  anti-LATS1 (#3477)  anti-p-LATS AL (#9157) | 1:1000  1:1000  1:1000 | Cell Signaling Technology  Cell Signaling Technology  Cell Signaling Technology | |
|  | anti-p-LATS HM (#8654) | 1:1000 | Cell Signaling Technology | |
|  | anti-YAP (#8418) | 1:1000 | Cell Signaling Technology | |
|  | anti-p-YAP (#4911) | 1:1000 | Cell Signaling Technology | |
|  | anti-STK25 (25821-1-AP) | 1:750 | Proteintech | |
|  | anti-NOX2 (19013-1-AP) | 1:1000 | Proteintech | |
|  | anti-iNOS (18985-1-AP) | 1:1000 | Proteintech | |
|  | anti-TNFα (sc-52746) | 1:100 | Santa Cruz Biotechnology | |
|  | anti-αSMA (ab5694) | 1:500 | Abcam | |
|  | anti-TGFβ (sc-130348) | 1:100 | Santa Cruz Biotechnology | |
| Secondary antibody | Alexa Fluor-488-labeled anti-rabbit IgG (A11008) | 1:500 | Invitrogen | |
|  | Alexa Fluor-488-labeled anti-mouse IgG (A21202) | 1:500 | Invitrogen | |
|  | Alexa Fluor-594-labeled anti-rat IgG (A11007) | 1:500 | Invitrogen | |
|  | Alexa Fluor-594-labeled anti-rabbit IgG (A21207) | 1:500 | Invitrogen | |
|  | Alexa Fluor-594-labeled anti-mouse IgG (A11005) | 1:500 | Invitrogen | |
|  | anti-rabbit IgG (#7074) | 1:1000 | Cell Signaling Technology | |
|  | anti-mouse IgG (#7076) | 1:1000 | Cell Signaling Technology | |
