# Supplementary material for: Silencing of STE20-type kinase TAOK1 confers protection against hepatocellular lipotoxicity through metabolic rewiring
Source: Hepatol Commun. 2023 Mar 17;7(4):e0037. doi: 10.1097/HC9.0000000000000037 (PMC10027040; doi:10.1097/HC9.0000000000000037)
Supplement: Supplementary file 3 [file hc9-7-e0037-s003.docx]

**SUPPORTING MATERIALS**

**Assessment of Lipid Metabolism**

To measure β-oxidation, cells were incubated in the presence of (9,10-^3^H[N])palmitic acid (PerkinElmer, Waltham, MA), and [^3^H]-labeled water was assessed as the product of free fatty acid oxidation [1]. Triacylglycerol (TAG) secretion and incorporation of media-derived [^3^H]glucose and [^3^H]oleic acid (both from PerkinElmer) into TAGs were determined as previously described [2]. Fatty acid uptake was quantified using the Quencher-Based Technology Fatty Acid Uptake Assay Kit (Molecular Devices, San Jose, CA). The TAG hydrolase activity was measured using [^3^H]triolein (PerkinElmer) as the substrate [3].

**Measurement of Glucose Metabolism**

Glycogen levels were assessed in immortalized human hepatocytes (IHHs) using the Glycogen Assay Kit (Sigma-Aldrich, St. Louis, MO). To measure hepatic glycogenolysis, IHHs were incubated in Dulbecco's Modified Eagle's Medium (DMEM) without glucose, glutamine, or pyruvate but containing 0.24 mmol/l 3-isobutyl-1-methylxanthine for 30 minutes; glucose content in the medium was then measured using the Amplex Red Glucose/Glucose Oxidase Assay Kit (Invitrogen, Carlsbad, CA). To determine the rate of gluconeogenesis, IHHs were cultured in glucose production assay medium (glucose- and phenol red-free DMEM containing 1 mmol/l sodium pyruvate, 20 mmol/l sodium lactate, and 15 mmol/l HEPES) for 4 hours; glucose content in the medium was then measured as described above.

To evaluate glycolysis, IHHs were transfected in the Seahorse XF Cell Culture Microplate (Agilent Technologies, Santa Clara, CA) as described above. The next day, culture medium was changed to the XF Base Medium (Agilent Technologies) supplemented with 10 mmol/l glucose, 1 mmol/l sodium pyruvate, and 2 mmol/l glutamine for 1 hour. Glycolytic flux was measured before and after injecting a mixture of rotenone (0.5 μmol/l; a complex I inhibitor) and antimycin A (0.5 μmol/l; a complex III inhibitor) followed by an injection of 2-deoxy-D-glucose (0.5 μmol/l), using the Seahorse XFe96 Extracellular Flux Analyzer (Agilent Technologies). Basal glycolysis and compensatory glycolysis were calculated using the Seahorse Glycolysis Rate Assay Report Generator (Agilent Technologies). Uptake of radiolabeled glucose into the IHHs was assessed in the presence of insulin as described earlier [2].

**Evaluation of Migration, Invasion, and Epithelial-Mesenchymal Transition**

To examine migratory capacity, HepG2-NTCP cells were added to the upper chambers of transwells with 8 µm pore size (Polycarbonate Cell Culture Inserts in Multi-Well Plates; Costar, Kennebunk, ME) and medium with 10% FBS was added in the bottom chambers for a chemotactic gradient. After a 24-hour incubation, cells on the upper surface of the membrane were removed using cotton swabs, and the cells on the bottom side of the membrane were stained with 0.1% crystal violet (Sigma-Aldrich). To measure invasive capacity, the transwells were coated with Matrigel matrix (Corning, Bedford, MA) before the experiment. Stained images were acquired using a Zeiss Axio Observer microscope with the ZEN Blue software and the crystal violet-labeled area was quantified in 6 randomly selected microscopic fields (×20) per well of the cell culture chamber using the ImageJ software. HepG2-NTCP cells were also processed for immunofluorescence with anti-N-cadherin or anti-E-cadherin antibodies (see Supplementary Table S1 for antibody information) to assess epithelial-mesenchymal transition (EMT).

**Quantitative Real-time PCR**

RNA was isolated from tissue samples and cultured human hepatocytes with the RNeasy Lipid Tissue Mini Kit (Qiagen, Hilden, Germany) or the EZNA Total RNA Kit (Omega Bio-Tek, Norcross, GA), and the following cDNA synthesis was performed using the High-Capacity cDNA Reverse Transcription Kit (Thermo Fisher Scientific, Waltham, MA). Relative quantification was performed with the QuantStudio 6 Flex Real-Time PCR System (Thermo Fisher Scientific) or the CFX Connect Real-Time System (Bio-Rad, Hercules, CA). Relative quantities of target transcripts were calculated from duplicate samples after normalization of the data to the endogenous control, 18S rRNA (Thermo Fisher Scientific).

**REFERENCES**

[1] Amrutkar M, Kern M, Nunez-Duran E, Stahlman M, Cansby E, Chursa U, et al. Protein kinase STK25 controls lipid partitioning in hepatocytes and correlates with liver fat content in humans. Diabetologia 2016;59:341-353.

[2] Amrutkar M, Cansby E, Nunez-Duran E, Pirazzi C, Stahlman M, Stenfeldt E, et al. Protein kinase STK25 regulates hepatic lipid partitioning and progression of liver steatosis and NASH. FASEB J 2015;29:1564-1576.

[3] Caputo M, Cansby E, Kumari S, Kurhe Y, Nair S, Stahlman M, et al. STE20-Type Protein Kinase MST4 Controls NAFLD Progression by Regulating Lipid Droplet Dynamics and Metabolic Stress in Hepatocytes. Hepatol Commun 2021;5:1183-1200.
